# Cancer Burden Variations and Convergences in Globalization: A Comparative Study on the Tracheal, Bronchus, and Lung (TBL) and Liver Cancer Burdens Among WHO Regions from 1990 to 2019

**DOI:** 10.1007/s44197-023-00144-x

**Published:** 2023-08-28

**Authors:** Mengwei Zhang, Weiqiu Jin, Yu Tian, Hongda Zhu, Ningyuan Zou, Yunxuan Jia, Long Jiang, Jia Huang, Yingjie Hu, Qingquan Luo

**Affiliations:** 1 Shanghai Lung Cancer Center, Shanghai Chest Hospital, Shanghai Jiao Tong University, Shanghai, China; 2 School of Medicine, Shanghai Jiao Tong University, Shanghai, China; 3 Department of Thoracic Surgery, Shanghai Chest Hospital, Shanghai Jiao Tong University, Shanghai, China

**Keywords:** Liver cancer, Lung cancer, Global burden of disease, Incidence, Death, DALY

## Abstract

Lung cancer and liver cancer are the leading and third causes of cancer death, respectively. Both lung and liver cancer are with clear major risk factors. A thorough understanding of their burdens in the context of globalization, especially the convergences and variations among WHO regions, is useful in precision cancer prevention worldwide and understanding the changing epidemiological trends with the expanding globalization. The Global Burden of Disease (GBD) and WHO Global Health Observatory (GHO) database were analyzed to evaluate the burden metrics and risk factors of trachea, bronchus, and lung (TBL) cancer and liver cancer. Western Pacific Region (WPR) had the highest age-standardized incidence rate (ASIR) for both liver cancer (11.02 [9.62–12.61] per 100,000 population) and TBL cancer (38.82 [33.63–44.04] per 100,000 population) in 2019. Disability-adjusted life years (DALYs) for liver and TBL cancer elevated with the increasing sociodemographic index (SDI) level, except for liver cancer in WPR and TBL cancer in European Region (EUR). Region of the Americas (AMR) showed the biggest upward trends of liver cancer age-standardized rates (ASRs), as well as the biggest downward trends of TBL cancer ASRs, followed by Eastern Mediterranean Region (EMR). Alcohol use and smoking were the leading cause of liver and TBL cancer death in most WHO regions. Variances of ASRs for liver and TBL cancer among WHO memberships have been decreasing during the past decade. The homogenization and convergence of cancer burdens were also demonstrated in different agegroups and sexes and in the evolution of associated risk factors and etiology. In conclusion, our study reflects the variations and convergences in the liver and lung cancer burdens among the WHO regions with the developing globalization, which suggests that we need to be acutely aware of the global homogeneity of the disease burden that accompanies increasing globalization, including the global convergences in various populations, risk factors, and burden metrics.

**Graphical Abstract:**

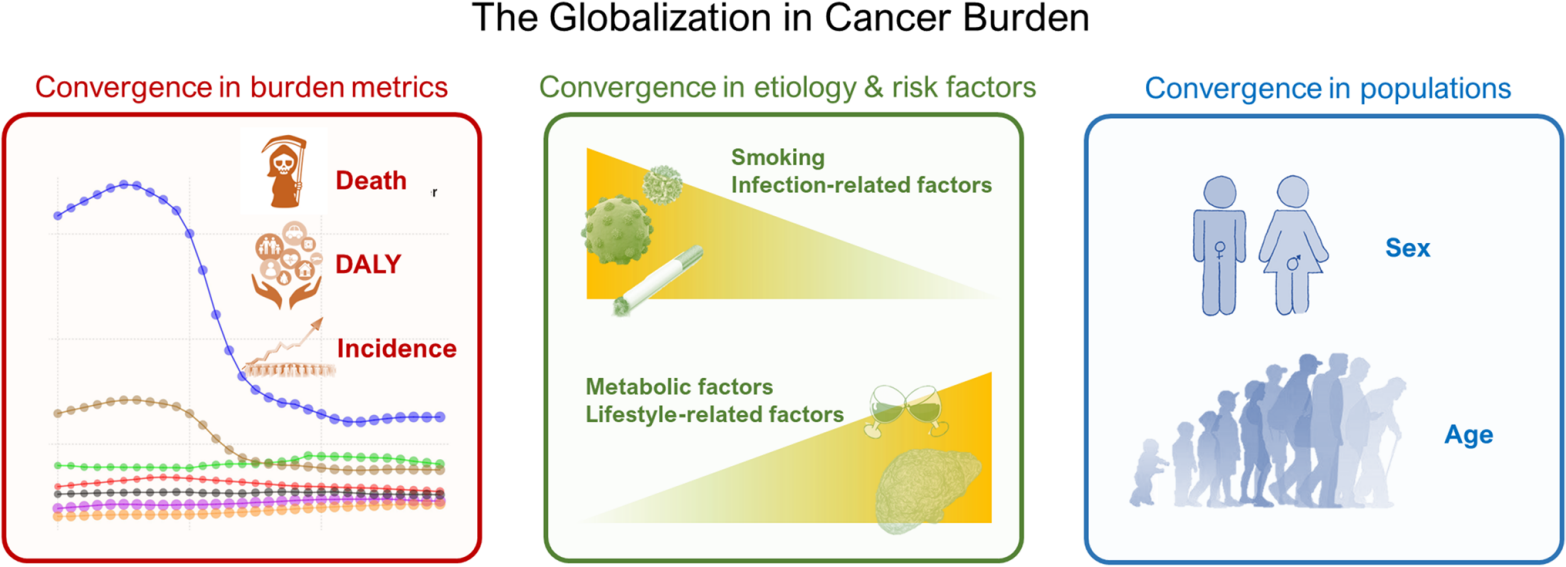

**Supplementary Information:**

The online version contains supplementary material available at 10.1007/s44197-023-00144-x.

## Background

Cancer is a major global public health issue. Globalization is tightly associated with the changing epidemiology of cancers. Both lung cancer and liver cancer are important species of human cancers. The epidemiologies of liver and lung cancers have undergone considerable changes as a result of growing economic interdependence, social integration, global unification of lifestyles, and other globalization-related factors.

Lung cancer is the second most common cancer and the leading cause of cancer death, which accounts for 11.4% (2,206,771) of the total cancer cases and 18.0% (1,796,144) of the total cancer deaths in 2020 [1]. Though the morbidity and mortality of liver cancer have decreased in many countries in recent years due to the control of hepatitis B virus (HBV) infections, which is the leading cause of liver cancer, as well as hepatitis C virus (HCV) and aflatoxin exposure, the huge cardinal number and the newly-developing risk factors (like metabolic diseases) make it still in a very high position in cancers, with an estimated 4.7% (905,677) of all new cancer cases and 8.3% (830,180) of cancer deaths in 2020 [1, 2]. Lung cancer and liver cancer are two of the few cancers whose major causes are clear and definite. It is traditionally thought that tobacco is the most important risk factor for lung cancer, though others are also proposed (such as air pollution) [3–6]. The risks of liver cancer are diverse and the major risks can change among different areas. In China, HBV infection plays a vital role in liver cancers, whereas in Western countries, the main cause of liver cancer is HCV infection [1, 2]. Though the major risk factors of liver cancer may be different among different regions, hepatitis virus infection is still the leading cause of liver cancer globally and metabolic risk factors (such as non-alcoholic steatohepatitis (NASH)) are definite and growing risk factors for liver cancer [2].

There are many similarities between liver cancer and lung cancer. For example, both lung cancer and liver cancer are more common in women than in men, although the reasons for them were likely to be different. The burden of lung cancer in males is roughly 2–4 times higher than in females, consistent with lung cancer being the leading cause of morbidity and mortality among men rather than women [7]. However, the trend among sexes in lung cancer seems different, a Global Burden of Disease (GBD) study showed that the age-standardized incidence rate (ASIR) decreased among males while increasing among females [7]. These differences may mainly come from the fact that smoking prevalence among women began later than men, despite other factors like genetic differences are also considered [8–10]. Similar to lung cancer, liver cancer is two to three times more common in men than in women, of which the reason is generally accepted that female hormone has a protective effect [1, 11–14]. Recently, screening methods have improved a lot for both lung and liver cancer. Low-dose computed tomography (LDCT) is routinely used to screen for lung cancer, which could diagnose more early-stage lung cancer compared with conventional radiography [15–17]. The screening for liver cancer becomes easier with the widespread and convenient application of liver ultrasonography and the serum marker alpha-fetoprotein (AFP) [2]. Critical progress in treatment has also been made in lung and liver cancer, both of which are represented by immunotherapy [18, 19]. Despite the improvement in diagnosis and treatment, the 5-year relative survival rate is only 22.9% for lung cancer and 20.8% for liver cancer in the United States from 2012 to 2018 according to National Cancer Institute's SEER database (https://seer.cancer.gov/statfacts/). Therefore, there is still a long way to control these two cancers.

Although trade and migration have been eternal phenomena in the human world, along with the accelerating globalization in recent years, the cancer burdens and their risk factors have been undergoing an unprecedented evolution, during which the metabolism factors, western diet, addictive items, medications, and hazardous and waste products are becoming emerging global risk factors in cancer public health [20]. A comparative study of the two in the context of globalization is of great significance and interest. First, a thorough understanding of the burden of lung cancer and liver cancer, especially the variations between countries and regions, is useful in uncovering the heterogeneity in the global burden of cancer and assisting precision prevention worldwide. Second, by investigating the risk factors for liver and lung cancer in different regions, it is possible to reveal the evolution of multiple classical cancer risk factors in the context of globalization, which has been highlighted in several previous researches [21, 22].

In our study, these comparisons are particularly based on the six World Health Organization (WHO) regions due to the WHO’s important role in the globalization of public health. Herein, we analyzed the burden of different WHO regions, sociodemographic index (SDI), ages, sexes, and risk factors in lung and liver cancer using the data from the GBD database from 1990–2019 in 204 (194 in WHO regions) countries and territories worldwide. In particular, we attempted to find the similarities and compare the difference between the ASIR, age-standardized death rate (ASDR), disability-adjusted life year (DALY), and their variation trends among these two cancers, which is necessary and helpful to control liver and lung cancer holistically and improve the global health. Meanwhile, we analyzed the evolution of lung and liver cancer burdens and their attributable risk factors with an emphasis on the trend of convergence hidden in the statistics, which revealed the globalized cancer burden and risk factors, a new phenomenon demonstrated by the convergences of lifestyle and human behaviour-related attributable risks and deserving further attention and keep-going observation in global health.

## Methods

### Data Sources and Software

The data used in this study were collected from GBD 2019 database, which is available from GHDx (website: https://vizhub.healthdata.org/gbd-results, last accessed on March 15, 2023) [23]. GBD 2019 estimated the burden of 369 diseases and injuries in 204 countries and territories with 87 risk factors, where the incidences, mortalities, and DALYs of TBL and liver cancers from 1990 to 2019 by sex, age, location, and risk factor were accessible. The vaccination coverage data used in this study were available at the WHO immunization data (website: http://immunizationdata.who.int/pages/coverage/hepb.html, accessed on July 28, 2023), where represented administrative and official Hepatitis B vaccination coverage (3rd dose) reported annually through the WHO/UNICEF Joint Reporting Form on Immunization (JRF). A detailed explanation of the immunization coverage estimation methods is provided in Ref. [24]. The global age-standardized body mass index (BMI) among adults and tobacco control data were accessed from the Global Health Observatory (GHO) data repository, a WHO's gateway to health-related statistics for its 194 Member States (website: https://www.who.int/data/gho/data/indicators/indicator-details/GHO, accessed on February 16, 2023). The analyses in this research were completed with MATLAB 9.8.0.1323502 (R2020a), and the visualization of results was performed by MATLAB 9.8.0.1323502 (R2020a) and GraphPad Prism 9.0.0.121.

### Selection of Regions

WHO is the authority responsible for public health within the United Nations system, which plays an essential role in improving local health systems and coordinating the global response to health threats. WHO Member States are grouped into 6 regions, and each region has a regional office: WHO Africa, WHO Americas, WHO Eastern Mediterranean, WHO Europe, WHO South-East Asia, and WHO Western Pacific. Our study incorporates national data within each region for analysis with reference to the official information given by WHO. For a detailed list of countries, please refer to Supplementary Materials Section 1.

### ASIR, ASDR, DALY, and Their EAPC Values

The EAPC values were calculated in this study. Assuming that the natural logarithm of incidence, death cases, or DALY varies linearly with time, EAPC could be calculated from two formulas:* Y* = *α* + *βX* + *ε* (*Y* = ln (incidence/death cases/DALY), *X* = calendar year, and *ε* = error term), and EAPC = 100 × (e^*β*^ − 1). Furthermore, the 95% CIs were also calculated according to the linear model. The trends of incidence, death, and DALY were reflected in EAPC values. Specifically, positive EAPCs and positive 95% CI are corresponding to an uptrend, while the downtrend of incidence, death, and DALY were reflected in negative EAPCs and 95% CI. The SDIs of different regions and countries were calculated in GBD 2019. To analyze the incidence, death, and DALY by sex in different age groups, we collected the data on both sexes of 20 age groups (1–4 years, 5–9 years, 10–14 years, 15–19 years, 20–24 years, 25–29 years, 30–34 years, 35–39 years, 40–44 years, 45–49 years, 50–54 years, 55–59 years, 60–64 years, 65–69 years, 70–74 years, 75–79 years, 80–84 years, 85–89 years, 90–94 years, and 95 + years).

### Risk Factors

We selected death and DALY-related risk factors of different levels covering all risk factors in GBD 2019 (refer to the list given in Supplementary Materials Section 2 for the specific risk factors included in this study), and finally obtained five risk factors related to liver cancer and seven risk factors related to TBL cancer. The obtained risk factors include alcohol use, drug use, high body-mass index, high fasting plasma glucose, and smoking for liver cancer; diet low in fruits, high fasting plasma glucose, occupational carcinogens, particulate matter pollution, residential radon, secondhand smoke, and smoking for TBL cancer.

## Results

### The Burdens of Tracheal, Bronchus, and Lung (TBL) and Liver Cancer During 1990–2019 in the World and WHO Members

The global incident cases of TBL cancer in all WHO members were 2,251,248.71 (2,059,474.81–2,442,036.11) in 2019, which increased 2.0 times compared with incident cases in 1990 (1,119,757.70 [1,073,432.81–1,172,185.19]). The global incident cases of liver cancer in all WHO members were 532,119.45 (484,371.12–586,210.12) in 2019, which increased 1.4 times compared with the incident cases in 1990 (371,847.88, [334,414.32–413,988.35]). The number of deaths in all WHO members increased by 91.77% caused by TBL cancer (from 1,060,916.3 [1,015,194.49–1,112,904.72] in 1990 to 2,034,466.28 [1,870,943.24–2,184,656.09] in 2019) and 32.67% caused by liver cancer (from 363,676.79 [328,435.94–404,191.48] in 1990 to 482,472.34 [442,231.44–523,630.96] in 2019.

The global ASIR, ASDR, and DALY of TBL cancer in all WHO members changed − 2.53%, − 7.72%, and + 0.6908% from 1990 (ASIR: 28.37 [27.16–29.65] per 100,000 population, ASDR: 27.28 [26–28.57] per 100,000 population, DALY: 27,022,284.98 [25,760,682.82–28,394,529.38]) to 2019 (ASIR: 27.65 [25.28–29.98] per 100,000 population, ASDR: 25.17 [23.16–27.01] per 100,000 population, DALY: 45,688,973.6 [42,144,896.66–49,177,438.15]), respectively. The global ASIR, ASDR, and DALY of liver cancer in all WHO members changed − 27.45%, − 33.37%, and + 11.05% from 1990 (ASIR: 8.97 [8.08–9.96] per 100,000 population, ASDR: 8.92 [8.07–9.9] per 100,000 population, DALY: 11,236,522.17 [10,020,011.24–12,632,864.72]) to 2019 (ASIR: 6.51 [5.94–7.16] per 100,000 population, ASDR: 5.94 [5.44–6.43] per 100,000 population, DALY: 12,477,953.81 [11,355,858.21–13,632,011.62]), respectively **(**Table 1**)**.

**Table 1:** The ASIR, ASDR, and DALY of liver and TBL cancer in 1990 and 2019 in WHO regions

WHO regions	WHO region (95% CI)	African region (95% CI)	Western Pacific region (95% CI)	Region of the Americas (95% CI)	European region (95% CI)	South-East Asia region (95% CI)	Eastern Mediterranean region (95% CI)
ASIR^a^							
Liver cancer (1990)	8.97 (8.08–9.96)	4.28 (3.65–5.31)	21.74 (18.83–25.06)	2.5 (2.41–2.56)	3.19 (3.1–3.26)	3.83 (3.47–4.21)	5.9 (4.94–6.83)
Liver cancer (2019)	6.51 (5.94–7.16)	4.08 (3.63–4.57)	11.02 (9.62–12.61)	3.92 (3.45–4.44)	4.39 (3.94–4.9)	4.04 (3.52–4.63)	6.45 (5.32–7.9)
Change/%	− 0.2746 (− 0.3729 to − 0.1565)	− 0.0461 (− 0.2441 to 0.1498)	− 0.493 (− 0.5832 to − 0.3738)	0.5718 (0.3786–0.7806)	0.3752 (0.2416–0.5273)	0.0541 (− 0.1153 to 0.2433)	0.0929 (− 0.1645 to 0.5007)
TBL cancer (1990)	28.37 (27.16–29.65)	9.9 (8.34–11.32)	29.94 (27–33.01)	41.02 (39.83–41.77)	37.79 (36.95–38.43)	10.63 (9.65–11.82)	11.85 (10.21–13.76)
TBL cancer (2019)	27.65 (25.28–29.98)	10.04(8.83–11.36)	38.82 (33.63–44.04)	29.96 (26.86–33.37)	32.69 (29.65–35.97)	11.28 (9.6–12.55)	13.59 (11.94–15.41)
Change/%	− 0.0253 (− 0.1238 to 0.0649)	0.0148 (− 0.1089 to 0.1705)	0.2964 (0.0828–0.552)	− 0.2696 (− 0.3451 to − 0.1904)	− 0.135 (− 0.2127 to − 0.0502)	0.0616 (− 0.1668 to 0.2345)	0.1473 (− 0.0532 to 0.405)
ASDR^b^							
Liver cancer (1990)	8.92 (8.07–9.9)	4.57 (3.89–5.7)	21.49 (18.76–24.73)	2.48 (2.38–2.55)	3.2 (3.09–3.27)	4.07 (3.67–4.49)	6.21 (5.19–7.2)
Liver cancer (2019)	5.94 (5.44–6.43)	4.37 (3.9–4.87)	9.5 (8.31–10.78)	3.61 (3.33–3.88)	3.95 (3.67–4.19)	4.22 (3.67–4.84)	6.49 (5.33–7.91)
Change/%	− 0.3337 (− 0.4182 to − 0.2316)	− 0.0444 (− 0.244 to 0.1398)	− 0.5579 (− 0.6376 to − 0.4565)	0.4591 (0.35–0.5576)	0.2321 (0.1647–0.3046)	0.0371 (− 0.1241 to 0.2185)	0.0459 (− 0.2045 to 0.4324)
TBL cancer (1990)	27.28 (26–28.57)	10.58 (8.96–12.1)	29.6 (26.61–32.81)	36.24 (35.05–36.95)	35.86 (35–36.49)	11.31 (10.13–12.57)	12.55 (10.84–14.59)
TBL cancer (2019)	25.17 (23.16–27.01)	10.77 (9.49–12.19)	34.73 (30.18–39.56)	25.67 (24.05–26.8)	29.05 (27.43–30.49)	11.87 (10.13–13.31)	14.27 (12.65–16.15)
Change/%	− 0.0773 (− 0.1591 to 0.0023)	0.0182 (− 0.1065 to 0.1668)	0.173 (− 0.0182 to 0.3963)	− 0.2916 (− 0.321 to − 0.2635)	− 0.1899 (− 0.2258 to − 0.1527)	0.0492 (− 0.1702 to 0.2232)	0.1372 (− 0.0625 to 0.3939)
DALY^c^							
Liver cancer (1990)	11,236,522.17 (10,020,011.24–12,632,864.72)	354,674.51 (300,106.5–428,094.53)	8,456,345.73 (7,282,599.18–9,874,112.31)	389,404.68 (378,625.14–398,236.09)	810,970.19 (791,860.93–825,979.89)	883,758.38 (812,222.32–965,547.03)	341,368.69 (296,013.94–386,801.79)
Liver cancer (2019)	12,477,953.81 (11,355,858.21–13,632,011.62)	679,194.69 (581,273.26–780,097.51)	6,705,036.27 (5,793,446.36–7,739,833)	1,071,815.24 (990,217.9–1,151,210.12)	1,345,088.82 (1,260,422.75–1,427,882.75)	1,887,854.47 (1,639,476.46–2,169,437.74)	788,964.32 (631,977.75–979,661.18)
Change/%	0.1105 (− 0.0495 to 0.3074)	0.915 (0.5617–1.3612)	− 0.2071 (− 0.3648 to 0.0009)	1.7524 (1.5364–1.9578)	0.6586 (0.5642–0.7606)	1.1362 (0.8102–1.5032)	1.3112 (0.7739–2.0964)
TBL cancer (1990)	27,022,284.98 (25,760,682.82–28,394,529.38)	598,493.93 (508,057.35–701,305.49)	8,829,984.08 (7,825,619.45–9,953,497.66)	5,222,196.65 (5,111,310.59–5,303,926.58)	9,734,388.74 (9,536,845.29–9,896,355.02)	2,041,333.2 (1,850,479.86–2,267,097.85)	595,888.37 (519,195.59–685,203.68)
TBL cancer (2019)	45,688,973.6 (42,144,896.66–49,177,438.15)	1,265,641.45 (1,089,729.62–1,467,647.33)	20,668,288.95 (17,831,822.22–23,827,799.89)	6,982,072.63 (6,655,042.52–7,266,919.92)	10,264,399.66 (9,715,663.56–10,777,355.28)	4,944,451.4 (4,216,280.37–5,555,813.6)	1,564,119.52 (1,367,787.8–1,794,356.2)
Change/%	0.6908 (0.5312–0.8533)	1.1147 (0.8124–1.4515)	1.3407 (0.9175–1.8377)	0.337 (0.2865–0.3906)	0.0544 (0.0053–0.106)	1.4222 (0.9149–1.8192)	1.6249 (1.1581–2.2105)

^a^Per 100,000 population

^b^Per 100,000 population

^c^Unit: years

**Fig. 1:**
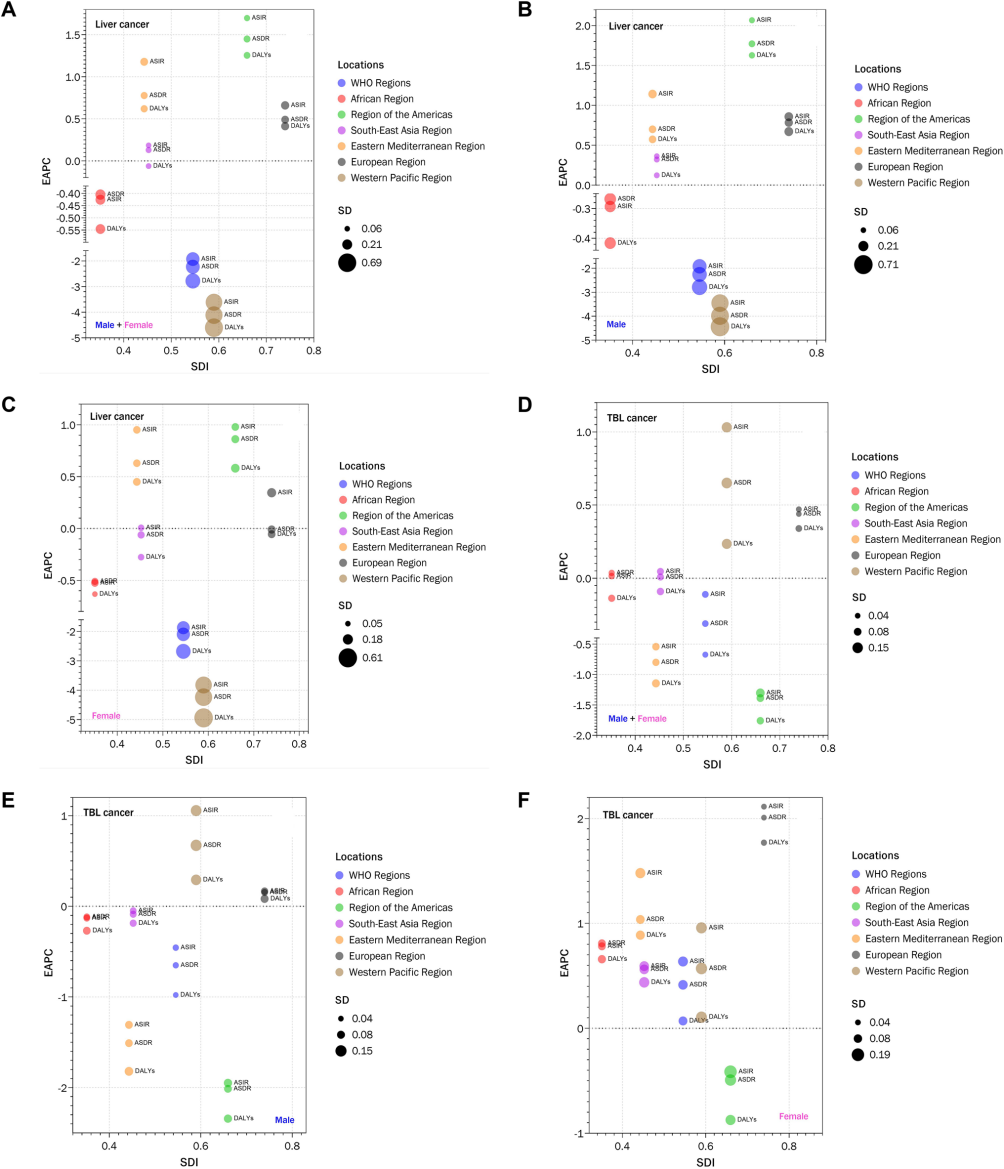
The EAPC of liver and TBL cancers by sexes in different WHO regions. The EAPC of liver cancer in both (**A**), male (**B**), and female (**C**). The EAPC of TBL cancer in both (**D**), male (**E**), and female (**F**)

Furthermore, estimated annual percentage changes (EAPCs), which describe the age-standardized rates (ASRs) or DALY trends, were analyzed among sexes. The ASIRs, ASDRs, and DALYs of liver cancer showed decreased trends in both sexes, males, and females (Fig. 1a–c). For TBL cancer, though decreased trends of ASIRs, ASDRs, and DALYs were also found among males, as well as among both sexes, the ASIRs, ASDRs, and DALYs increased among females (Fig. 1d–f).

**Fig. 2:**
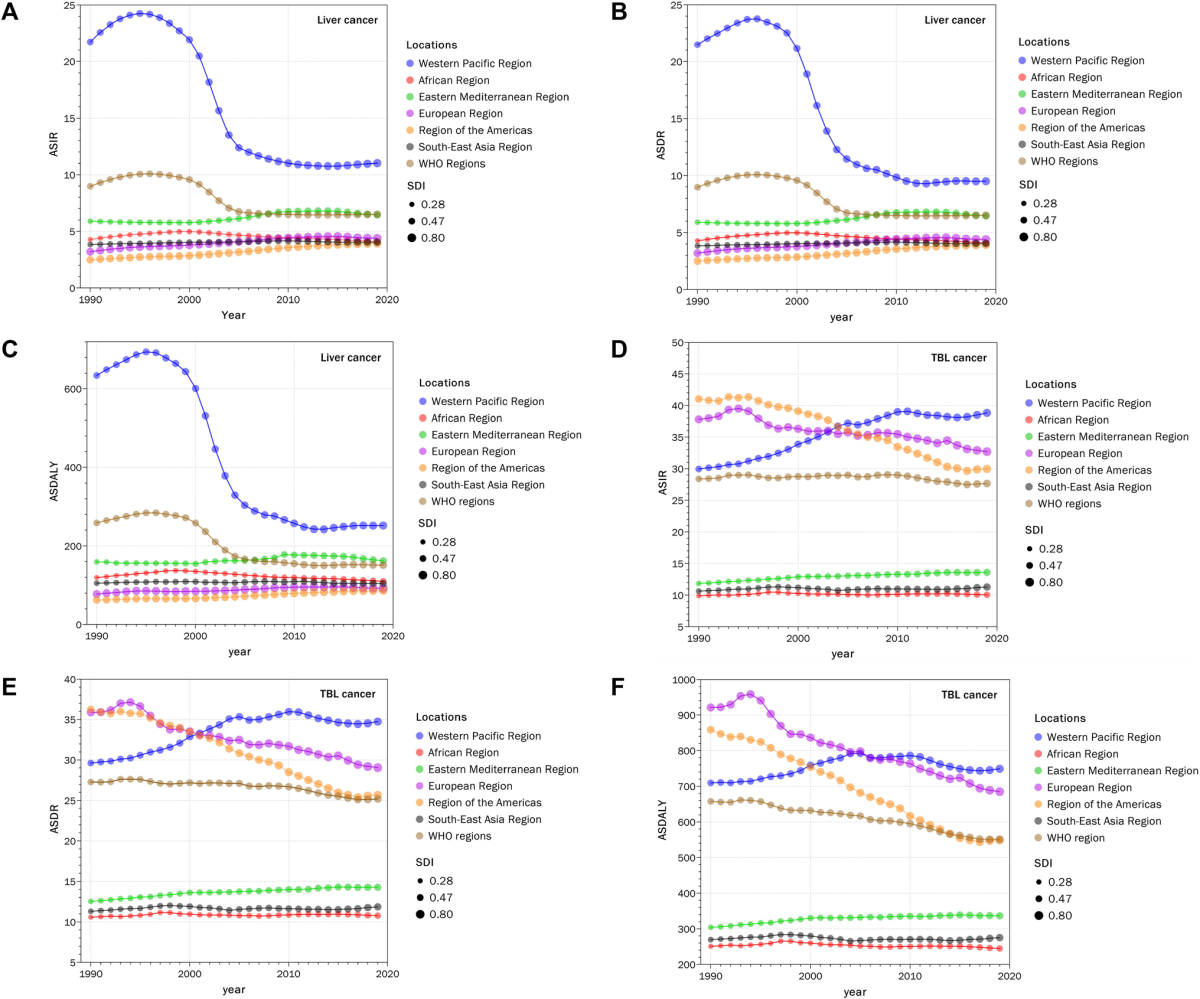
The ASIR, ASDR, and DALY of liver cancer and TBL cancer among WHO regions based on SDI. ASIR (**A**), ASDR (**B**), and ASDALY (**C**) of liver cancer. ASIR (**D**), ASDR (**E**), and ASDALY (**F**) of TBL cancer

The burden of liver and TBL cancer had different change patterns from 1990 to 2019 globally. ASIR, ASDR, and ASDALY of liver cancer in all WHO members rose slowly from 1990 to 1996, declined rapidly after 1996, and stabilized gradually since 2005, while the ASIR and ASDR of TBL cancer kept declining slightly (Fig. 2). The patterns of absolute DALY numbers for liver and TBL cancer were also different. The DALY of liver cancer in all WHO members increased to 14,221,535.34 (13,357,980.31–15,084,693.45) by 1999 before decreasing to only 9,852,003.49 (9,419,983.55–10,309,344.12) by the end of 2006, while the DALY of TBL cancer kept rising from 1990 to 2019 (Supplementary Materials Section 3 Fig. S3E–S3F). Additionally, the global trends of liver and TBL cancer burdens in GBD regions were similar to the trends in all WHO members (Supplementary Materials Section 3 Fig. S3).

### The Regional Burdens of Liver and TBL Cancer During 1990–2019 Among WHO Regions

Regions with the highest ASIR for liver cancer were Western Pacific Region (WPR) with 21.74 (18.83–25.06) per 100,000 population in 1990 and 11.02 (9.62–12.61) per 100,000 population in 2019 while Region of the Americas (AMR) had lowest ASIR (2.5 [2.41–2.56] per 100,000 population in 1990 and 3.92 [3.45–4.44] per 100,000 population in 2019). Regions with the highest ASIR for TBL cancer changed from AMR with 41.02 (39.83–41.77) per 100,000 population in 1990 to WPR with 38.82 (33.63–44.04) per 100,000 population in 2019 while African Region (AFR) had the lowest ASIR from 1990 to 2019 (9.9 [8.34–11.32] per 100,000 population in 1990 and 10.04 [8.83–11.36] per 100,000 population in 2019) (Table 1, Fig. 2).

The pattern of ASDR for liver and TBL cancer was the same as ASIR. The highest ASDR of liver cancer was 21.49 [18.76–24.73] per 100,000 population in 1990 and 9.5 [8.31–10.78] per 100,000 population in 2019 in WPR and the region with the lowest ASDR was AMR (2.48 [2.38–2.55] per 100,000 population in 1990 and 3.61 [3.33–3.88] per 100,000 population in 2019). Eastern Mediterranean Region (EMR) ranked second both in ASIR and ASDR of liver cancer from 5.9 (4.94–6.83) per 100,000 population in 1990 to 6.45 (5.32–7.9) per 100,000 population in 2019 (ASIR) and from 6.21 (5.19–7.2) per 100,000 population in 1990 to 6.49 (5.33–7.91) per 100,000 population in 2019 (ASDR) (Table 1, Fig. 2).

The ASDR of TBL cancer changed from 1990 to 2019 with the highest ASDR in AMR in 1990 (36.24 [35.05–36.95] per 100,000 population) and WPR in 2019 (34.73 [30.18–39.56] per 100,000 population) while the lowest ASDR was 10.58 [8.96–12.1] per 100,000 population in 1990 and 10.77 [9.49–12.19] per 100,000 population in 2019 in AFR. European Region (EUR) ranked the second both in ASIR and ASDR of TBL cancer from 37.79 (36.95–38.43) per 100,000 population in 1990 to 32.69 (29.65–35.97) per 100,000 population in 2019 (ASIR) and from 35.86 (35–36.49) per 100,000 population in 1990 to 29.05 (27.43–30.49) per 100,000 population in 2019 (ASDR) (Table 1, Fig. 2).

Globally, the ASIR and ASDR of liver and TBL cancer decreased from 1990 to 2019. However, no individual WHO regions shared the same pattern with the whole WHO region in both liver and TBL cancer. WPR and AFR showed a decline in the ASIR and ASDR of liver cancer from 1990 to 2019, while the ASIR and ASDR of TBL cancer increased. On the contrary, the ASIR and ASDR of liver cancer in AMR and EUR increased from 1990 to 2019, while the ASIR and ASDR of TBL cancer decreased. As for South-East Asia Region (SEAR) and EMR, the ASIR and ASDR of liver and TBL cancer both increased (Fig. 2a, b, d, e).

Regionally, DALYs for both liver and TBL cancer among different WHO regions elevated with increasing SDI values, except for liver cancer in WPR, which decreased during 1998–2011 and kept increasing since 2011, and TBL cancer in EUR, which showed decrement during 1994–1998 and remained relatively stable since 1998 (Supplementary Materials Section 3 Fig. S3E–S3F). To be more specific, the DALY of liver cancer in WPR was maximized in 1998 (10,788,373.44 [9,952,918.76–11,734,761.94]) and minimized in 2011 (5,500,567.741 [5,059,173.687–6,038,431.186]). Similarly, the DALY of TBL cancer in EUR was maximized in 1994 (10,530,241.56 [10,360,471.59–10,729,756.08]) and minimized in 1998 (9,710,468.76 [9,527,655.09–9,856,903.44]). Furthermore, among the six WHO regions, WPR ranked in the top one on DALY in both liver and TBL cancer, while the following second was SEAR in liver cancer and EUR in TBL cancer.

Further analysis of EAPC values found that the trends of ASRs and DALYs were different among different WHO regions. AMR showed the biggest upward trends of liver cancer ASRs, as well as the biggest downward trends of TBL cancer ASRs, followed by EMR. On the contrary, the biggest downward trends of liver cancer ASRs and the biggest upward trends of TBL cancer ASRs were found in WPR. Notably, in EUR, upward trends of ASRs and DALYs were found in both liver and TBL cancer (Fig. 1a, d).

Notably, the ASIR and ASDR in WPR and AMR changed in opposite directions. Among WHO regions, WPR had the fastest decline in the ASIR (− 49.30%) and ASDR (− 55.79%) of liver cancer, as well as the fastest increase in ASIR (+ 29.64%) and ASDR (+ 17.30%) of TBL cancer. On the contrary, AMR decreased fastest in TBL cancer (ASIR: − 26.96%, ASDR: − 29.16%) and increased fastest in liver cancer (ASIR: + 57.18%, ASDR: + 45.91%). Moreover, it seems that the ASIR and ASDR of liver and TBL cancer in WPR since 2010 were approaching that in AMR in the 1990s. More specifically, the highest ASRs of TBL cancer were found in AMR in the 1990s while that was found in WPR since 2010 with similar values. Though the highest ASRs of liver cancer remained in WPR from 1990 to 2019, the values are getting closer since 2010. The variation tendencies of liver and TBL cancer between WPR and AMR indicated that WPR might go through the same experience that AMR has experienced. The levels and trends of ASRs in EUR were found nearly the same as that in the AMR.

Different patterns of these trends were also found among different sexes. For example, the upward trend of ASR for liver cancer among males in AMR was significantly larger than that in EMR, while the gap was relatively small among females (Fig. 1b, c). Among males, only WPR and EUR showed upward trends of ASRs for TBL cancer, while most of the WHO regions among females showed upward trends except AMR, where the largest downward trends were found regardless of sex (Fig. 1e, f).

### The Epidemiological Trends of Liver and TBL Cancer During 1990–2019 at the National/Territorial Level

**Fig. 3:**
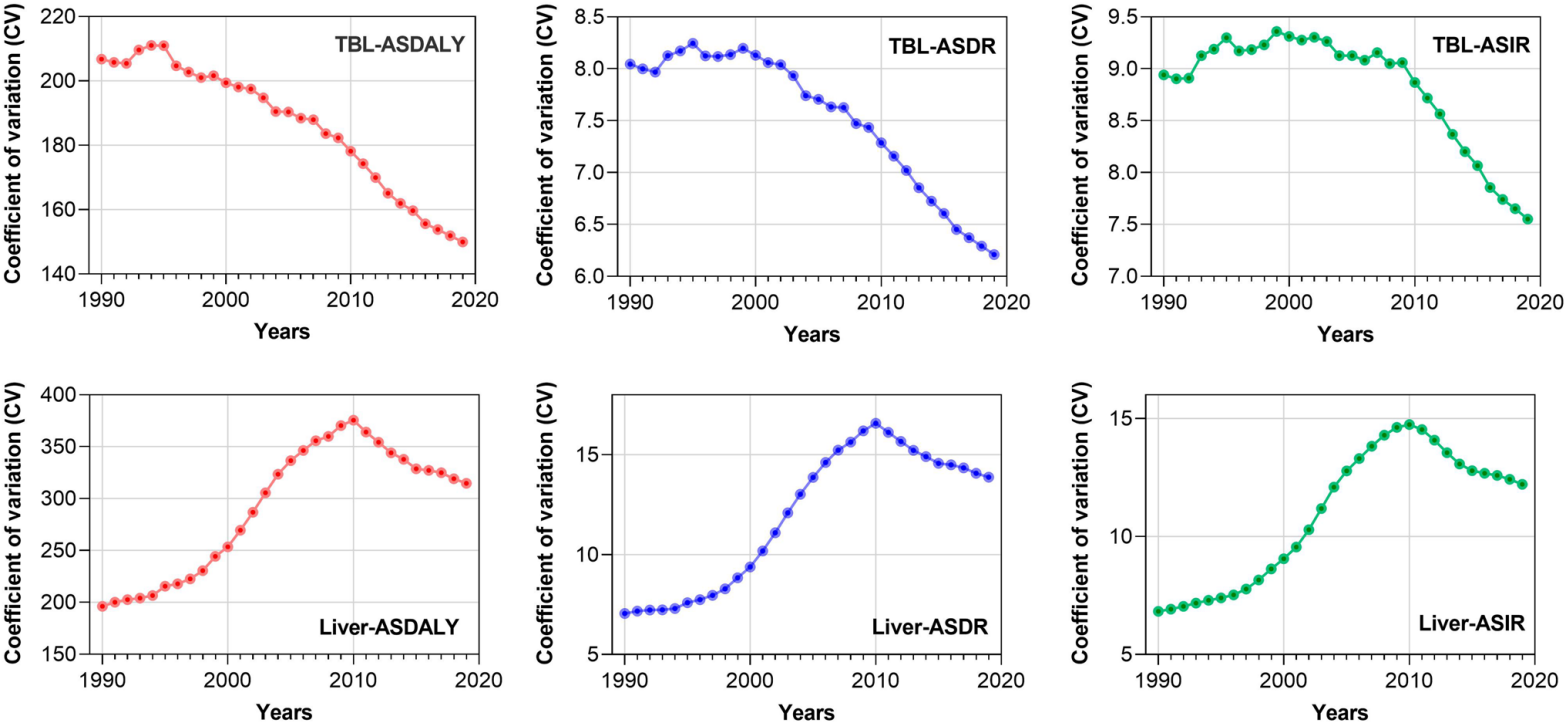
The variances of ASIRs, ASDALYs, and ASDRs for TBL and liver cancer among WHO memberships

In 2019, the ASIR and ASDR for liver cancer were highest in Mongolia (ASIR: 105.22 [82.57–131.46] per 100,000 population, change: + 0.64 [0.22–1.21]; ASDR: 115.23 [91.48–142.48] per 100,000 population, change: + 0.73 [0.3–1.3]), Gambia (ASIR: 38.21 [27.53–49.67] per 100,000 population, change: + 0.28 (− 0.15 to 0.86); ASDR: 39.51 [29.01–50.99] per 100,000 population, change: + 0.28 [− 0.14 to 0.84]), and Guinea (ASIR: 32.17 [22.33–41.9] per 100,000 population, change: + 0.08 (− 0.28 to 0.49); ASDR: 34.05 [23.98–44.01] per 100,000 population, change: + 0.07 [− 0.29 to 0.48]) (Table 2, Supplementary Materials Section 4). Figure 3 demonstrates the variances of ASIRs, ASDALYs, and ASDRs for TBL and liver cancer among WHO memberships. It can be seen that their trends are similar. TBL cancer remained flat or increased slightly in the global variances of the three main cancer burden indicators between 1990 and 2000, but after 2000, these variances showed a consistent and steady decline. Liver cancer, on the other hand, showed increases in the variances of disease burden metrics between 1990 and 2010 and a steady decrease in the last decade. These convergent trends in the variances of ASIRs, ASDALYs, and ASDRs for TBL and liver cancer among WHO member countries in recent years may be attributed to the globalization of effective public health interventions, lifestyle modifications, and advancements in medical care.

**Table 2:** Top ten countries/territories with the most cancer burdens

Rank	1	2	3	4	5	6	7	8	9	10
Liver cancer										
1990 ASIR										
Country/territories	Mongolia	Guinea	Gambia	China	Tonga	Thailand	Egypt	Qatar	Mali	Democratic People's Republic of Korea
ASIR^a^ (95% CI)	64.22 (51.72–77.51)	29.91 (24.86–35.83)	29.75 (22.26–39.05)	25.71 (21.73–30.35)	23.33 (16.2–30.14)	20.81 (18.13–23.74)	16.75 (13.1–20.2)	16.14 (12.14–21.04)	15.12 (12.43–18.25)	14.62 (11.35–18.97)
Change (95% CI)	0.64 (0.22–1.21)	0.08 (− 0.28 to 0.49)	0.28 (− 0.15 to 0.86)	− 0.59 (− 0.68 to − 0.48)	0.04 (− 0.25 to 0.44)	0.16 (− 0.16 to 0.6)	0.25 (− 0.17 to 0.94)	0.08 (− 0.25 to 0.54)	− 0.04 (− 0.31 to 0.28)	− 0.3 (− 0.51 to − 0.04)
2019 ASIR										
Country/territories	Mongolia	Gambia	Guinea	Tonga	Thailand	Republic of Korea	Egypt	Eswatini	Qatar	Honduras
ASIR^a^ (95% CI)	105.22 (82.57–131.46)	38.21 (27.53–49.67)	32.17 (22.33–41.9)	24.33 (17.65–31.9)	24.18 (17.89–32.01)	22.8 (18.72–27.32)	20.92 (15.09–28.51)	18.43 (5.61–32.97)	17.39 (12.83–22.91)	14.8 (6.8–21.7)
Change (95% CI)	0.64 (0.22–1.21)	0.28 (− 0.15 to 0.86)	0.08 (− 0.28 to 0.49)	0.04 (− 0.25 to 0.44)	0.16 (− 0.16 to 0.6)	1.07 (0.57–1.74)	0.25 (− 0.17 to 0.94)	2.27 (− 0.4 to 6.71)	0.08 (− 0.25 to 0.54)	0.34 (0.02–0.95)
1990 ASDR										
Country/territories	Mongolia	Guinea	Gambia	China	Tonga	Thailand	Egypt	Qatar	Mali	Democratic People's Republic of Korea
ASDR^a^ (95% CI)	66.77 (54.18–79.9)	31.8 (26.71–38.1)	30.76 (23.19–40.07)	25.99 (22.29–30.55)	23.93 (16.74–30.82)	21.6 (18.83–24.52)	17.43 (13.67–20.93)	17.37 (12.99–22.54)	15.7 (12.98–18.83)	14.77 (11.51–19.02)
Change (95% CI)	− 0.64 (− 0.71 to − 0.53)	− 0.31 (− 0.52 to − 0.06)	− 0.39 (− 0.53 to − 0.22)	− 0.15 (− 0.38 to 0.19)	− 0.07 (− 0.25 to 0.16)	− 0.32 (− 0.54 to − 0.02)	0.08 (− 0.22 to 0.45)	− 0.23 (− 0.49 to 0.15)	0.46 (0.01 to 1.02)	− 0.35 (− 0.52 to − 0.05)
2019 ASDR										
Country/territories	Mongolia	Gambia	Guinea	Tonga	Thailand	Egypt	Eswatini	Republic of Korea	Honduras	Qatar
ASDR^a^ (95% CI)	115.23 (91.48–142.48)	39.51 (29.01–50.99)	34.05 (23.98–44.01)	24.74 (18.09–32.04)	24.01 (17.88–31.65)	21.25 (15.44–28.92)	19.09 (5.98–33.88)	16.2 (14.47–17.94)	16.14 (7.41–23.5)	15.88 (11.76–20.79)
Change (95% CI)	0.73 (0.3–1.3)	0.28 (− 0.14 to 0.84)	0.07 (− 0.29 to 0.48)	0.03 (− 0.25 to 0.42)	0.11 (− 0.19 to 0.52)	0.22 (− 0.18 to 0.89)	2.21 (− 0.39 to 6.45)	0.47 (0.16–0.86)	0.35 (0.04–0.98)	− 0.09 (− 0.37 to 0.3)
1990 ASDALY										
Country/territories	Mongolia	Gambia	Guinea	China	Tonga	Thailand	Egypt	Mali	Democratic People's Republic of Korea	Kiribati
ASDALY^a^ (95% CI)	1,726.2 (1,371.23–2,109.52)	876.47 (636.2–1170.09)	833.45 (689.56–1014.67)	769.11 (653.21–913.07)	655.95 (453.35–861.63)	575.32 (500.14–659.92)	457.68 (366.19–548.17)	445.32 (363.38–537.01)	437.07 (332.88–577.51)	423.71 (325.23–531.34)
Change (95% CI)	0.48 (0.08–1.04)	0.27 (− 0.15 to 0.91)	0.07 (− 0.3 to 0.49)	− 0.66 (− 0.73 to − 0.55)	0.04 (− 0.26 to 0.5)	0.07 (− 0.24 to 0.49)	0.18 (− 0.22 to 0.84)	− 0.08 (− 0.35 to 0.25)	− 0.31 (− 0.55 to − 0.02)	− 0.17 (− 0.41 to 0.15)
2019 ASDALY										
Country/territories	Mongolia	Gambia	Guinea	Tonga	Thailand	Egypt	Eswatini	Lesotho	Mali	Republic of Korea
ASDALY^a^ (95% CI)	2,558.12 (1,961.96–3,278.63)	1,116.06 (789.18–1,480.33)	889.94 (611.41–1163.89)	684.06 (487.15–913.98)	615.25 (450.05–818.04)	537.85 (382.38–742.42)	534.72 (151.32–1,000.58)	410.68 (162.44–658.81)	408.97 (298.64–547.03)	390.81 (348.58–435.93)
Change (95% CI)	0.48 (0.08–1.04)	0.27 (− 0.15 to 0.91)	0.07 (− 0.3 to 0.49)	0.04 (− 0.26 to 0.5)	0.07 (− 0.24 to 0.49)	0.18 (− 0.22 to 0.84)	2.45 (− 0.42 to 7.48)	1.91 (− 0.33 to 5.45)	− 0.08 (− 0.35 to 0.25)	0.31 (0.03–0.68)
TBL cancer										
1990 ASIR										
Country/territories	Greenland	United States of America	Northern Mariana Islands	Denmark	Canada	Belgium	Netherlands	United Kingdom	Hungary	Czechia
ASIR^a^ (95% CI)	85.59 (77.79–95.54)	58.87 (57.24–59.96)	55.9 (48.24–65.55)	52.86 (51.14–54.47)	52.15 (50.42–53.77)	51.59 (49.65–53.54)	50.22 (48.58–51.69)	49.95 (48.58–50.78)	49.27 (47.7–50.73)	48.77 (47.34–50.65)
Change (95% CI)	− 0.09 (− 0.26 to 0.08)	− 0.23 (− 0.34 to − 0.11)	− 0.2 (− 0.33 to − 0.05)	− 0.19 (− 0.37 to 0.02)	− 0.16 (− 0.35 to 0.06)	− 0.23 (− 0.4 to − 0.02)	− 0.11 (− 0.3 to 0.12)	− 0.2 (− 0.33 to − 0.05)	0.05 (− 0.14 to 0.28)	− 0.33 (− 0.45 to − 0.17)
2019 ASIR										
Country/territories	Greenland	Monaco	Montenegro	Hungary	Serbia	Brunei Darussalam	United States of America	Netherlands	Northern Mariana Islands	Palau
ASIR^a^ (95% CI)	77.71 (64.38–90.6)	75.57 (61.39–90.82)	56.72 (46.5–68.94)	51.94 (42.61–63.65)	49.38 (38.82–62.4)	46.15 (40.91–51.83)	45.13 (39.11–52.21)	44.82 (35.35–56.6)	44.82 (38.99–50.05)	44.54 (36.03–55.03)
Change (95% CI)	− 0.09 (− 0.26 to 0.08)	0.58 (0.19–1.1)	0.18 (− 0.06 to 0.48)	0.05 (− 0.14 to 0.28)	0.26 (− 0.03 to 0.66)	− 0.01 (− 0.21 to 0.24)	− 0.23 (− 0.34 to − 0.11)	− 0.11 (− 0.3 to 0.12)	− 0.2 (− 0.33 to − 0.05)	0.05 (− 0.23 to 0.37)
1990 ASDR										
Country/territories	Greenland	Northern Mariana Islands	Belgium	Denmark	United States of America	Brunei Darussalam	Hungary	Netherlands	Czechia	United Kingdom
ASDR^a^ (95% CI)	87.78 (79.76–97.23)	58.72 (50.72–68.68)	50.2 (48.43–51.89)	49.89 (48.36–51.26)	49.35 (47.82–50.29)	47.79 (39.36–56.55)	47.73 (46.29–49.08)	47.47 (46.06–48.78)	46.91 (45.6–48.57)	46.34 (45.05–47.1)
Change (95% CI)	− 0.11 (− 0.28 to 0.07)	− 0.21 (− 0.34 to − 0.07)	− 0.3 (− 0.34 to − 0.26)	− 0.26 (− 0.32 to − 0.2)	− 0.27 (− 0.3 to − 0.24)	− 0.06 (− 0.25 to 0.17)	0.01 (− 0.17 to 0.22)	− 0.2 (− 0.24 to − 0.15)	− 0.38 (− 0.49 to − 0.23)	− 0.29 (− 0.32 to − 0.26)
2019 ASDR										
Country/territories	Greenland	Monaco	Montenegro	Hungary	Northern Mariana Islands	Serbia	Palau	Brunei Darussalam	Poland	Nauru
ASDR^a^ (95% CI)	78.23 (63.91–91.97)	64.23 (52.32–76.9)	53.36 (43.82–64.43)	48.12 (39.58–58.44)	46.24 (40.35–51.51)	45.96 (36.13–57.51)	45.79 (37.28–56.29)	44.83 (39.66–50.19)	44.31 (36.96–52.61)	40.44 (27.06–52.79)
Change (95% CI)	− 0.11 (− 0.28 to 0.07)	0.46 (0.12–0.92)	0.16 (− 0.07 to 0.45)	0.01 (− 0.17 to 0.22)	− 0.21 (− 0.34 to − 0.07)	0.19 (− 0.08 to 0.57)	0.02 (− 0.24 to 0.33)	− 0.06 (− 0.25 to 0.17)	− 0.03 (− 0.2 to 0.15)	− 0.04 (− 0.28 to 0.26)
1990 ASDALY										
Country/territories	Greenland	Northern Mariana Islands	Hungary	Czechia	Poland	Denmark	Montenegro	United States of America	Belgium	Kazakhstan
ASDALY^a^ (95% CI)	2,174.56 (1,953.04–2,432.11)	1,350.27 (1,166.03–1,609.25)	1,295.93 (1,255.74–1,333.8)	1,238.29 (1,201.82–1,284.59)	1,224.74 (1,205.37–1,244.49)	1,218.32 (1,184.29–1,250.34)	1,205.26 (1,052.86–1,353.47)	1,197.78 (1,172.11–1,216.85)	1,185.72 (1,148.32–1,224.87)	1,175.24 (1,116.63–1,238.9)
Change (95% CI)	− 0.19 (− 0.35 to − 0.01)	− 0.24 (− 0.38 to − 0.1)	− 0.06 (− 0.24 to 0.15)	− 0.46 (− 0.57 to − 0.33)	− 0.14 (− 0.29 to 0.03)	− 0.35 (− 0.4 to − 0.3)	0.11 (− 0.12 to 0.4)	− 0.36 (− 0.38 to − 0.34)	− 0.33 (− 0.37 to − 0.29)	− 0.55 (− 0.62 to − 0.48)
2019 ASDALY										
Country/territories	Greenland	Monaco	Montenegro	Hungary	Serbia	Palau	Poland	Northern Mariana Islands	North Macedonia	Bosnia and Herzegovina
ASDALY^a^ (95% CI)	1,769 (1,436.88–2,101.49)	1,483.26 (1,181.87–1,811.68)	1,343.58 (1,092.16–1,633.36)	1,220.97 (992.75–1,496.03)	1,183.5 (919.2–1,497.59)	1,071.77 (855.72–1,337.81)	1,051.75 (866.5–1,258.55)	1,021.74 (877.24–1,161.75)	1,000.4 (760.47–1,285.24)	957.92 (736.31–1,213.42)
Change (95% CI)	− 0.19 (− 0.35 to − 0.01)	0.37 (0.01–0.83)	0.11 (− 0.12 to 0.4)	− 0.06 (− 0.24 to 0.15)	0.13 (− 0.14 to 0.5)	0.01 (− 0.26 to 0.34)	− 0.14 (− 0.29 to 0.03)	− 0.24 (− 0.38 to − 0.1)	0.26 (− 0.04 to 0.63)	− 0.03 (− 0.26 to 0.24)

^a^Per 100,000 population

Similarly, the top three countries/territories with the highest ASIR and ASDR for TBL cancer were the same as the countries/territories in 2019. The ASIR for TBL cancer was highest in Greenland (77.71 [64.38–90.6] per 100,000 population), Monaco (75.57 [61.39–90.82] per 100,000 population), and Montenegro (56.72 [46.5–68.94] per 100,000 population) in 2019 with a change of − 0.09 (− 0.26 to 0.08), + 0.58 (0.19–1.1), and + 0.18 (− 0.06 to 0.48) from 1990 to 2019. The ASDR for TBL cancer was also highest in Greenland (78.23 [63.91–91.97] per 100,000 population), Monaco (64.23 [52.32–76.9] per 100,000 population), and Montenegro (53.36 [43.82–64.43] per 100,000 population) in 2019, with a change of + 0.11 (− 0.28 to 0.07), + 0.46 (0.12–0.92), and + 0.16 (− 0.07 to 0.45) from 1990 to 2019 (Table 2, Supplementary Materials Section 4). Interestingly, the countries/territories with the highest ASIR, ASDR, and ASDALY among the 204 countries/territories for both liver and TBL cancer have not changed (liver cancer: Mongolia, TBL cancer: Greenland).

The percentage change of ASIR in liver or TBL cancer differed substantially between countries/territories from 1990 to 2019. The largest increases in ASIR were in Cabo Verde (+ 9.58 [7.4–12.48]), Uzbekistan (+ 5.96 [4.62–7.48]), and Armenia (+ 5.05 [3.92–6.3]) for liver cancer and Honduras (+ 0.68 [0.14–1.38]), Cabo Verde (+ 0.62 [0.24–1.01]), and Monaco (+ 0.58 [0.19–1.1]) for TBL cancer, while the largest decreases were found in Poland (− 0.72 [− 0.77 to − 0.66]), Saint Kitts and Nevis (− 0.71 [− 0.76 to − 0.64]), and Bermuda (− 0.7 [− 0.76 to − 0.63]) for liver cancer and Kyrgyzstan (− 0.54 [− 0.61 to − 0.48]), Bahrain (− 0.52 [− 0.65 to − 0.35]), and Kazakhstan (− 0.5 [− 0.57 to − 0.42]) for TBL cancer. As for ASDR, the percentage changes were also different among countries/territories, with Cabo Verde (+ 9.52 [7.42–12.43]), Uzbekistan (+ 6.04 [4.79–7.5]), and Armenia (+ 5.26 [4.17–6.46]) for liver cancer and Honduras (+ 0.67 [0.15–1.33]), Cabo Verde (+ 0.64 [0.25–1.03]), and Mozambique (+ 0.5 [0.08–1.01]) for TBL cancer showing the largest increases. By contrary, Bermuda (− 0.73 [− 0.78 to − 0.66]), Poland (− 0.73 [− 0.77 to − 0.68]), and Saint Kitts and Nevis (− 0.71 [− 0.76 to − 0.65]) for liver cancer and Kyrgyzstan (− 0.53 [− 0.6 to − 0.47]), Bahrain (− 0.52 [− 0.65 to − 0.35]), and Kazakhstan (− 0.49 [− 0.57 to − 0.42]) for TBL cancer showed the largest decreases.

### The Burden of Liver and TBL Cancer Among Age-Groups and Sexes

**Fig. 4:**
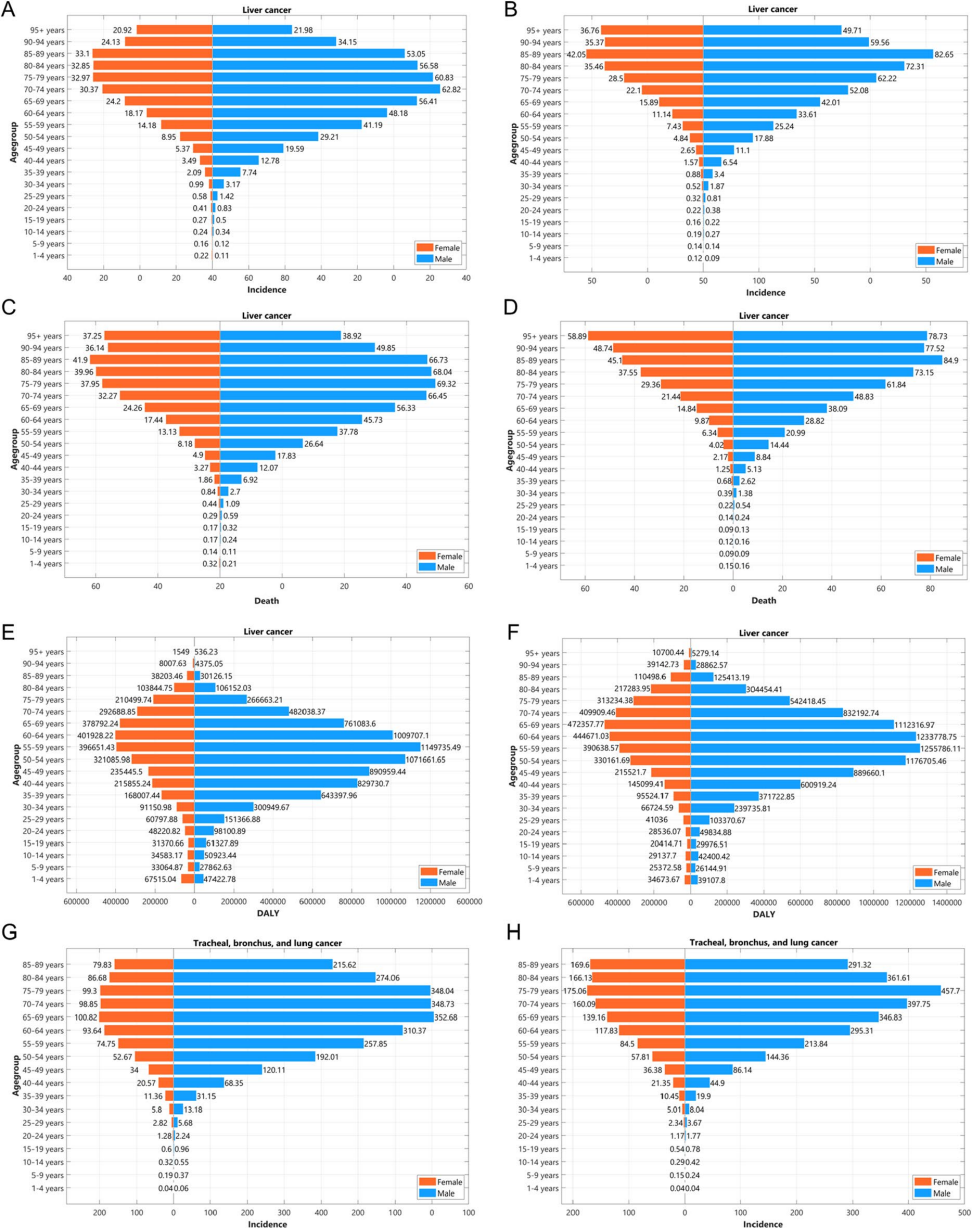

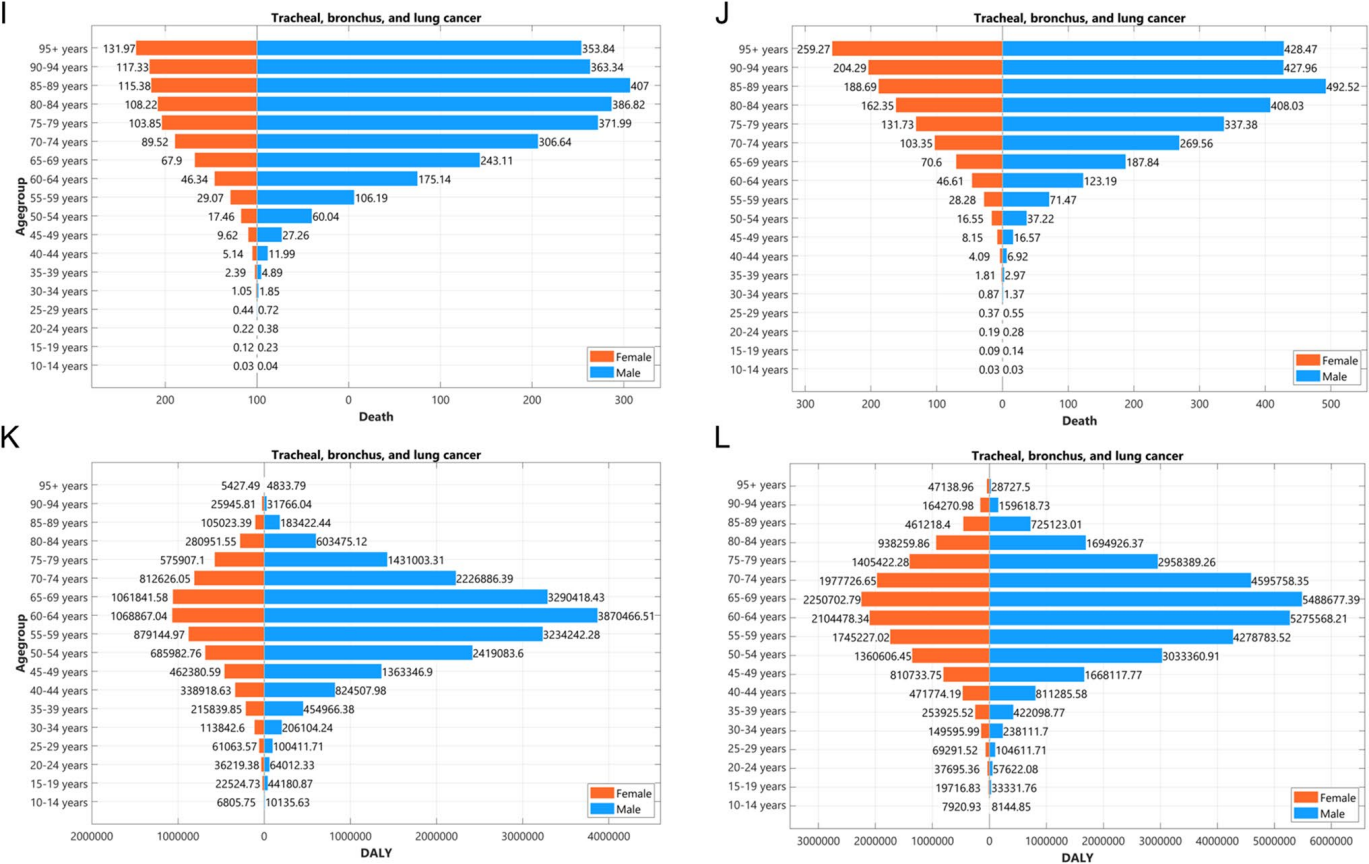
The incidence, death, and DALY of liver and TBL cancers in 1990 and 2019 by sexes in different agegroups. The incidence (per 100,000 population) of liver cancer in 1990 (**A**) and 2019 (**B**). The death (per 100,000 population) of liver cancer in 1990 (**C**) and 2019 (**D**). The DALY (per 100,000 population) of liver cancer in 1990 (**E**) and 2019 (**F**). The incidence (per 100,000 population) of TBL cancer in 1990 (**G**) and 2019 (**H**). The death (per 100,000 population) of TBL cancer in 1990 (**I**) and 2019 (**J**). The DALY (per 100,000 population) of TBL cancer in 1990 (**K**) and 2019 (**L**)

The incidences, deaths, and DALYs were also analyzed in a subgroup (Fig. 4, Supplementary Materials Section 5). The overall trend was that incidence and mortality rates for liver and TBL cancer increased with increasing age. However, there were some different details among the aged. In 2019, the incidence rates of liver cancer increased with the increasing age groups up to 85–89 years, after which the rates decreased again. Things were slightly different in 1990 when the incidence rates of liver cancer in males started to decrease after 70–74 years meaning a delayed peak age in males from 1990 to 2019, whereas decrement in females happened after 85–89 years, the same as in 2019 (Fig. 4a, b). The age groups for TBL cancer when the incidence rate peaked were also postponed in both males and females from 1990 to 2019 (from 65–69 years to 75–79 years) (Fig. 4g, h).

The incidence rate of TBL and liver cancer is highest among the aged population, rather than the oldest population. This can be attributed to the dose–response effect of risk factors. Prolonged exposure to viruses or inflammations is required for liver cell damage and carcinogenesis [25]. A similar dose–response relationship has been observed between smoking and lung cancer [26]. When individuals are exposed to these risks to a certain extent, cancer develops, leading to higher mortality rates among high-risk populations. In the case of individuals aged 90 + or 80 + , the proportion of individuals with a high risk or genetic predisposition to cancer may have decreased, resulting in a lower incidence of liver or TBL cancer.

From 1990 to 2019, the age at which lung cancer incidence peaked was delayed for both men and women. The age at which liver cancer incidence peaked was delayed in men but not in women, possibly because the age at which it peaked was already high in women. The delay in the peak of incidence among age groups indicates that there may have been changes in risk factors or advancements in medical interventions during this period that impacted the disease progression. In addition, the aging of hepatitis/smoking population may also contribute to the delay of the incidence peak for liver/TBL cancer.

The pattern of mortality rates for liver cancer in 1990 was the same as the incidence pattern in 1990. The mortality rates of liver cancer in females increased with the increasing age groups without decreasing in 2019, whereas the peak age group in males was postponed from 75–79 to 85–89 years (Fig. 4c, d). The pattern of mortality rates for TBL cancer was alike in 1990 and 2019 with increasing mortality rates by increasing age group in females and decreasing mortality in males after the peak at 85–89 years (Fig. 4i, j).

Generally, the highest DALYs of liver and TBL cancer were both concentrated at 55–69 years for both sexes in both 1990 and 2019 (Fig. 4e, f, k, l), which suggests that cancer screening for them is particularly important. For the DALYs in liver cancer, a relatively prominent peak was observed in the youngest age group (1–4 years) for both sexes in both 1990 and 2019 (Fig. 4e, f), which may be related to the fact that hepatoblastoma tends to occur in infants under 2 years old [27], and the young age causes larger DALYs [28].

In 2019, the male/female ratio of incident cases was 2.4 in liver cancer (374,822.66 [333,618.38–420,243.05] in males, 157,296.79 [139,926.16–175,383.97] in females) and 2.1 in TBL cancer (1,516,994.64 [1,368,976.84–1,669,952.83] in males, 734,254.07 [655,697.56–810,887.93] in females). The total burdens of liver or TBL cancer were higher in males than in females. The male/female ratio of incidence, mortality, and DALY in liver cancer remained stable from 1990 to 2019 with a ratio of 2.1–2.6 (2.37 for incidence, 2.19 for mortality, 2.60 for DALY in 2019 and 2.27 for incidence, 2.17 for mortality, 2.50 for DALY in 1990). However, TBL cancer showed a lower gap between males and females in 2019 compared with that in 1990 (ratio of male/female in 2019: incidence 2.05, deaths 2.10, DALYs 2.21; in 1990: incidence 2.80, deaths 2.84, DALYs 3.01), meaning the TBL cancer burden relatively increased in women from 1990 to 2019.

For both liver and TBL cancer, the male–female ratio of incident cases is close to 2:1. However, the causes for the low incidence in females are different: the protective effect of estrogen for liver cancer [12–14, 29] while the low rates of smoking in females for TBL cancer [30]. This difference also caused different male–female ratios in different age groups. The male/female ratio of incident cases for lung cancer does not change significantly within age groups (steady between 1.5 and 2.5). However, the male/female ratio of incident cases for liver cancer varies greatly in different age groups (up to 5 times) with a sharp inverted “V” shape among age groups. This difference may be related to the change in female estrogen levels during one’s lifetime [31, 32]. In addition, the ASRs of liver cancer decreased in both males and females. Though the downtrends of ASRs for TBL cancer were found among males, the ASRs among females were increasing, which were thought to be associated with not only smoking but also indoor air pollution, as well as other risk factors [33]. Therefore, we should attach more importance to women with a high risk of lung cancer and to postmenopausal women who are susceptible to liver cancer.

The incidence ratio of male/female for liver cancer showed an inverted V-shaped trend with age and peaked at the age of 45–49 years with a ratio of 4.18 in 2019 while the lowest ratio was 0.83 at 1–4 years. The incidence ratio of male/female in TBL cancer seemed more stable among different age groups ranging from 0.93 at the age of 10–14 years to 2.61 at the age of 85–89 years in 2019. In addition, the incidence, deaths, and DALYs of liver and TBL cancer in 1990 and 2019 by sex in different age groups among six WHO regions were shown in Supplementary Materials Section 5. Generally, the burdens of both liver and TBL cancer were higher in males than in females among different WHO regions though there remained differences in details. In 2019, the incidence rate for liver cancer in females increased with increasing age and peaked at 85–89 years after which declines were shown among AFR, EMR, EUR, AMR, and SEAR. However, among WPR, the incidence rate in females showed an increase in the 95 + age group (71.88 per 100,000 population) more than that in the 85–89 age group (69.77 per 100,000 population). The peaked age groups for the incidence rate of liver cancer in males among different WHO regions can be divided into two groups. The incidence rate of four regions (AFR, EMR, AMR, and SEAR) in males peaked at 80–84 age groups, which is earlier than that in females, while the incidence rate of two regions (EUR and WPR) in males peaked at 85–89 age groups, which is the same with that in females. The incidence ratio of male/female for liver cancer ranged from 1.7 to 2.7 among different WHO regions with the highest ratio in WPR (2.73) and the lowest ratio in AFR (1.79). As for TBL cancer, in 2019, the incidence rate in females increased with increasing age and peaked at 70–74 years among AFR and AMR, at 75–79 among EMR and EUR, and at 85–89 among SEAR and WPR. The peaked age groups for the incidence rate of TBL cancer in males among different WHO regions ranged from 65 to 79 years. The incidence rate in AFR and EMR for TBL cancer in males peaked at 65–69 age groups, while the incidence rate in males in EUR, AMR, SEAR, and WPR peaked at 70–79 age groups. Notably, the peaked age for TBL cancer incidence rate in males was later than that in females among AMR (female: 70–74 years; male: 75–79), which was different from that among other WHO regions. The incidence ratio of male/female for TBL cancer ranged from 1.3 to 3.2 among different WHO regions with the highest ratio in EMR (3.19) and the lowest ratio in AMR (1.30).

**Fig. 5:**
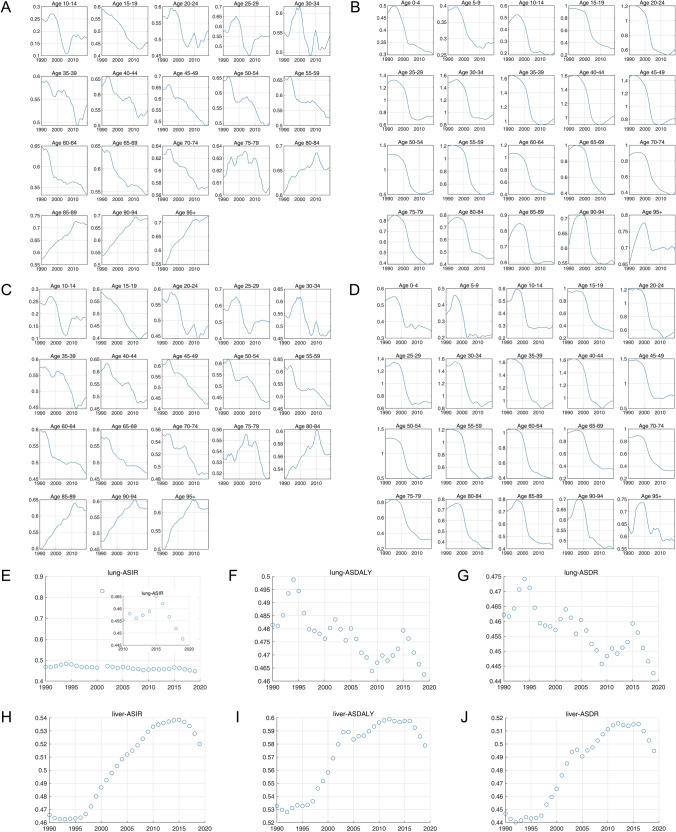
Variances of incidence rate, DALY rate, and their male-to-female ratios among six WHO regions. **A** Incidence rate variances of different age-groups among six WHO regions (TBL cancer). **B** Incidence rate variances of different age-groups among six WHO regions (liver cancer). **C** DALY rate variances of different age-groups among six WHO regions (TBL cancer). **D** DALY rate variances of different age-groups among six WHO regions (liver cancer). **E**–**G** The variances of male-to-female ratios in ASIRs, ASDALYs, and ASDRs for TBL cancer among WHO regions. **H**–**J** The variances of male-to-female ratios in ASIRs, ASDALYs, and ASDRs for liver cancer among WHO regions

Figure 5 demonstrates the variances of incidence rate and DALY rate among six WHO regions. From the Fig. 5a, b, it can be seen that in the most agegroups (especially the mid-age groups), the variances of incidence rate among six WHO regions have been decreasing in the past decades. Similar trends of DALY rate could be observed in Fig. 5c, d. The variances of male-to-female ratios in ASIRs, ASDALYs, and ASDRs of TBL and liver cancers among WHO regions are shown in Fig. 5e, j, where decreasing trends could be witnessed in the 5 years.

In summary, these findings highlight the importance of considering gender-specific, temporal, and spatial influences on cancer development and emphasize the need for further research to unravel the underlying mechanisms driving these trends.

### The Attributable Risks of Liver and TBL Cancer in 1990 and 2019 Among WHO Regions

**Fig. 6:**
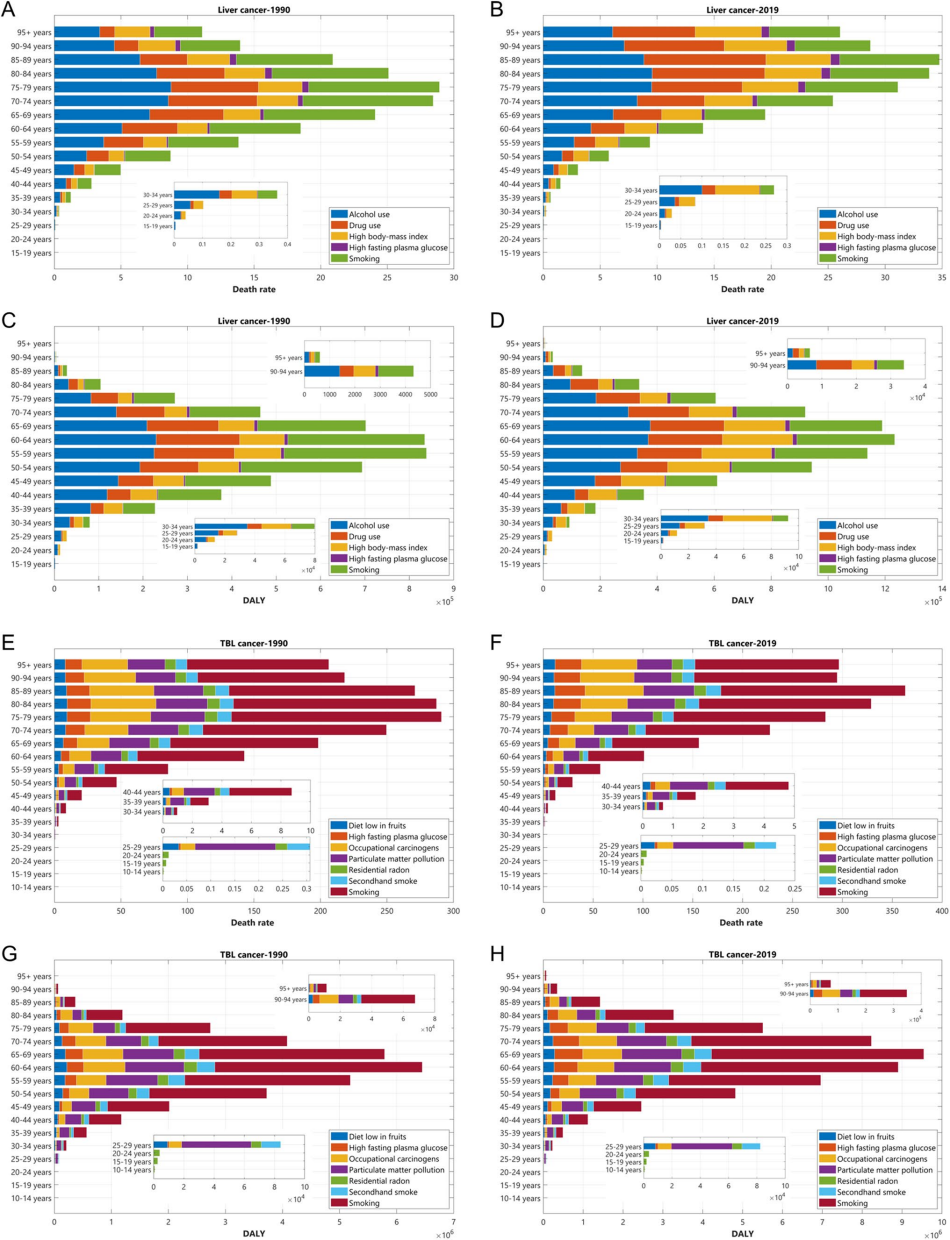
The death rate and DALY of liver and TBL cancers in 1990 and 2019 by risk factors. The death rate (per 100,000 population) of liver cancer in 1990 (**A**) and 2019 (**B**). The DALY (per 100,000 population) of liver cancer in 1990 (**C**) and 2019 (**D**). The death rate (per 100,000 population) of TBL cancer in 1990 (**E**) and 2019 (**F**). The DALY (per 100,000 population) of TBL cancer in 1990 (**G**) and 2019 (**H**)

Figure 6 and Supplementary Materials Sect. 6 show the risk factors attributed to death rates and DALYs of liver and TBL cancers in 1990 and 2019 in WHO regions. Though different percentages may exist in different WHO regions, alcohol use and smoking were the two risk factors with the highest percentages attributed to death rates and DALY of liver cancer, followed by drug use and high body-mass index, while high fasting plasma glucose contributed to the least. However, different risk factors may be with a particularly high or low contribution in a region compared with other WHO regions. For example, the contribution of drug use was far lower than the contribution of high body-mass index in AFR and EMR, while it was higher than the contribution of high body-mass index in remained four WHO regions.

Smoking kept the leading cause of death rates and DALYs of TBL cancer in all age groups ≥ 35 years, whereas at the age group of 25–39, the percentage of particulate matter pollution-related death rates and DALYs were highest compared with other risk factors and residential radon was the only factors that contribute to death rates and DALYs in the youngest age group (10–14 years, 15–19 years, and 20–24 years). Other risk factors contributing to TBL cancer included occupational carcinogens, high fasting plasma glucose, secondhand smoke, and diet low in fruits.

Alcohol use was also the leading cause of liver cancer death in AFR, EUR, AMR, and SEAR. However, the leading cause of liver cancer death in EMR was high body-mass index, which was also the second leading cause of liver cancer death in AFR, and in WPR was smoking, which was also the second leading cause of liver cancer death in EMR and SEAR. In addition, EUR, AMR, and WPR shared the same second cause (drug use) of liver cancer death. Notably, the leading cause of liver cancer death and DALY has not changed from 1990 to 2019 among different WHO regions except for EMR whose leading cause was smoking in 1990 and high body-mass index in 2019. Smoking was the leading cause of TBL cancer death rate in all six WHO regions in the past three decades. The second and third causes of TBL cancer death were particulate matter pollution and occupational carcinogens among AFR, EUR, SEAR, and WPR in 2019 and among all six WHO regions in 1990. However, the third cause of TBL cancer death in 2019 had changed to high fasting plasma glucose among AMR and EMR.

**Fig. 7:**
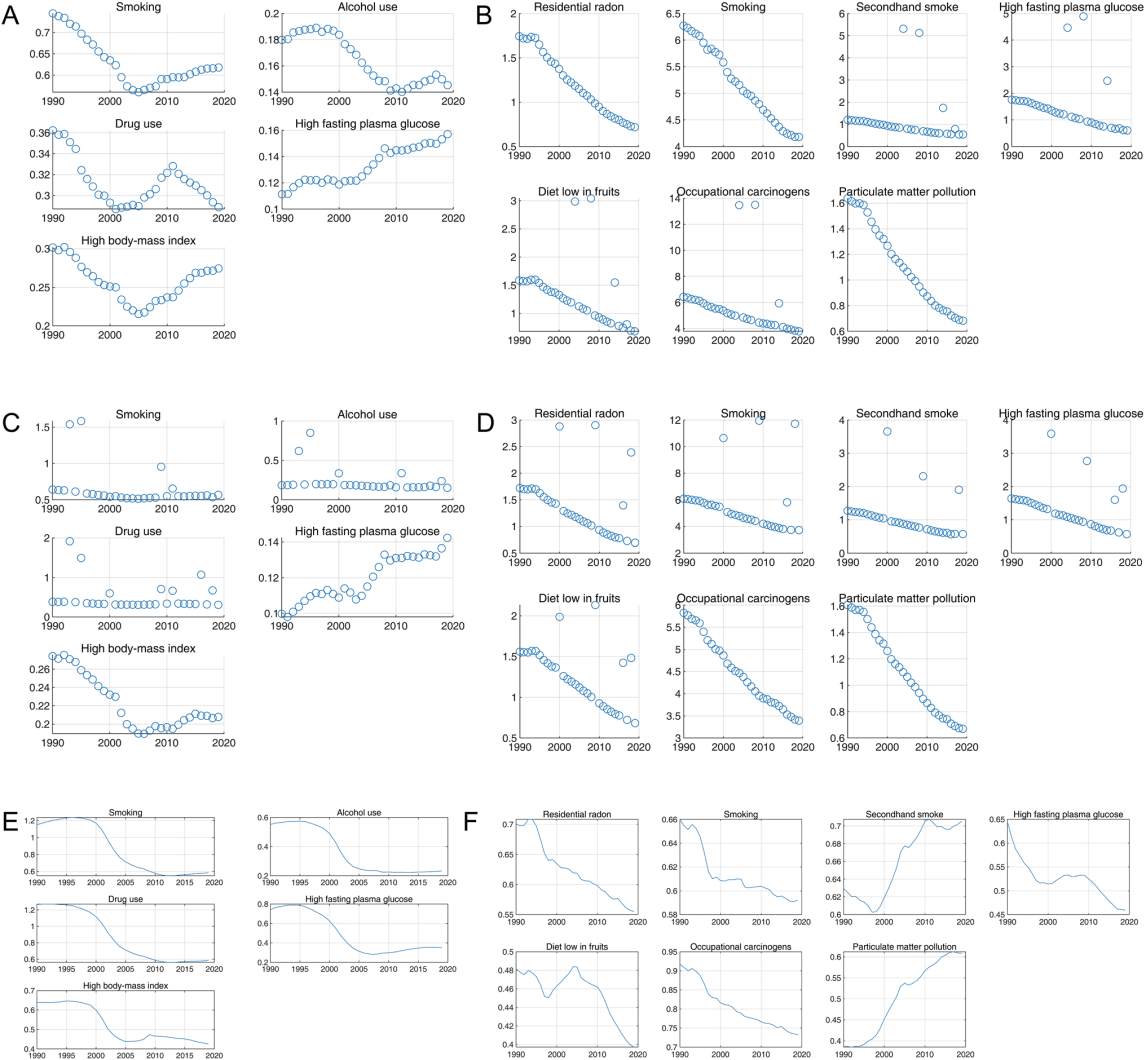
Variances in risk factors of liver and TBL cancers. **A** Variance of the male-to-female ratios of contribution from each risk factor to the ASDALY rate across the six WHO regions (liver cancer). **B** Variance of the male-to-female ratios of contribution from each risk factor to the ASDALY rate across the six WHO regions (TBL cancer). **C** Variance of the male-to-female ratios of contributions from each risk factor to the ASDR among six WHO regions (liver cancer). **D** Variance of the male-to-female ratios of contributions from each risk factor to the ASDR among six WHO regions (TBL cancer). **E** Variance of the contributions from individual risk factors to ASDALY rate among WHO member countries (liver cancer). **F** Variance of the contributions from individual risk factors to ASDALY rate among WHO member countries (TBL cancer)

Figure 7 demonstrates the variances in risk factors of liver and TBL cancers, including variances of the male-to-female ratios of contribution from each risk factor to the ASDALY and ASD rate across the six WHO regions in TBL and liver cancer (Fig. 7a, d) and variances of the contributions from individual risk factors to ASDALY rate among WHO member countries in liver and TBL cancer (Fig. 7e, f). The results showed that except for several risk factors (for example, the sex ratio of high fasting plasma glucose and high BMI to ASDALY rate in liver cancer), the variances in contributions to cancer burden metrics from most risk factors manifested declining trends.

**Fig. 8:**
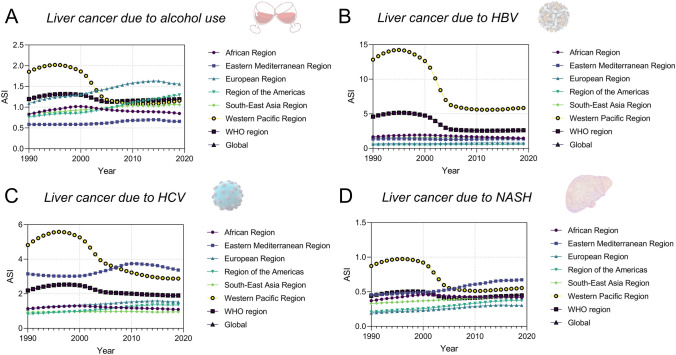
The globalization of the liver cancer etiology. Age-standardized incidences (ASIs) of liver cancer due to **A** alcohol use, **B** HBV, **C** HCV, **D** NASH

**Fig. 9:**
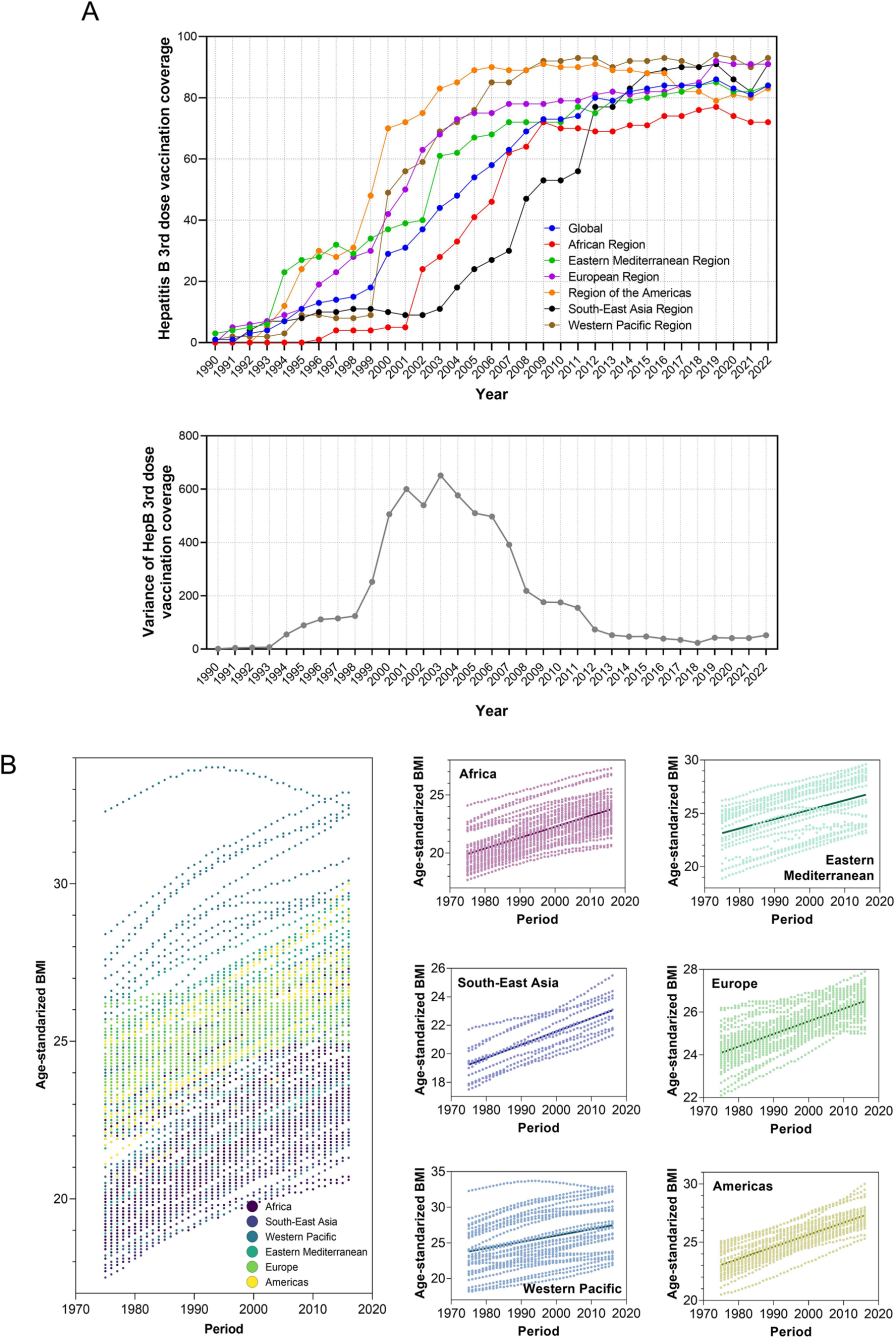
The globalization of the cancer etiology (vaccination and high BMI). **A** An example of protective factor (hepatitis B 3rd dose vaccination), where the upper figure shows the vaccination coverages among six WHO regions and the lower figure calculates the variances among six WHO regions according to their coverage statistics. **B** An example of risk factor (age-standardized BMI values), where linear regression relationships between BMI level and year were established for six WHO regions (point estimation and their 95% CI: AFR, 0.09401 [0.08886, 0.09916]; AMR, 0.1037 [0.09957, 0.1077]; EMR, 0.08777 [0.07614, 0.09940]; EUR, 0.05910 [0.05683, 0.06138]; SEAR, 0.09294 [0.08429, 0.1016]); WPR, 0.08932 [0.07200, 0.1066])

**Fig. 10:**
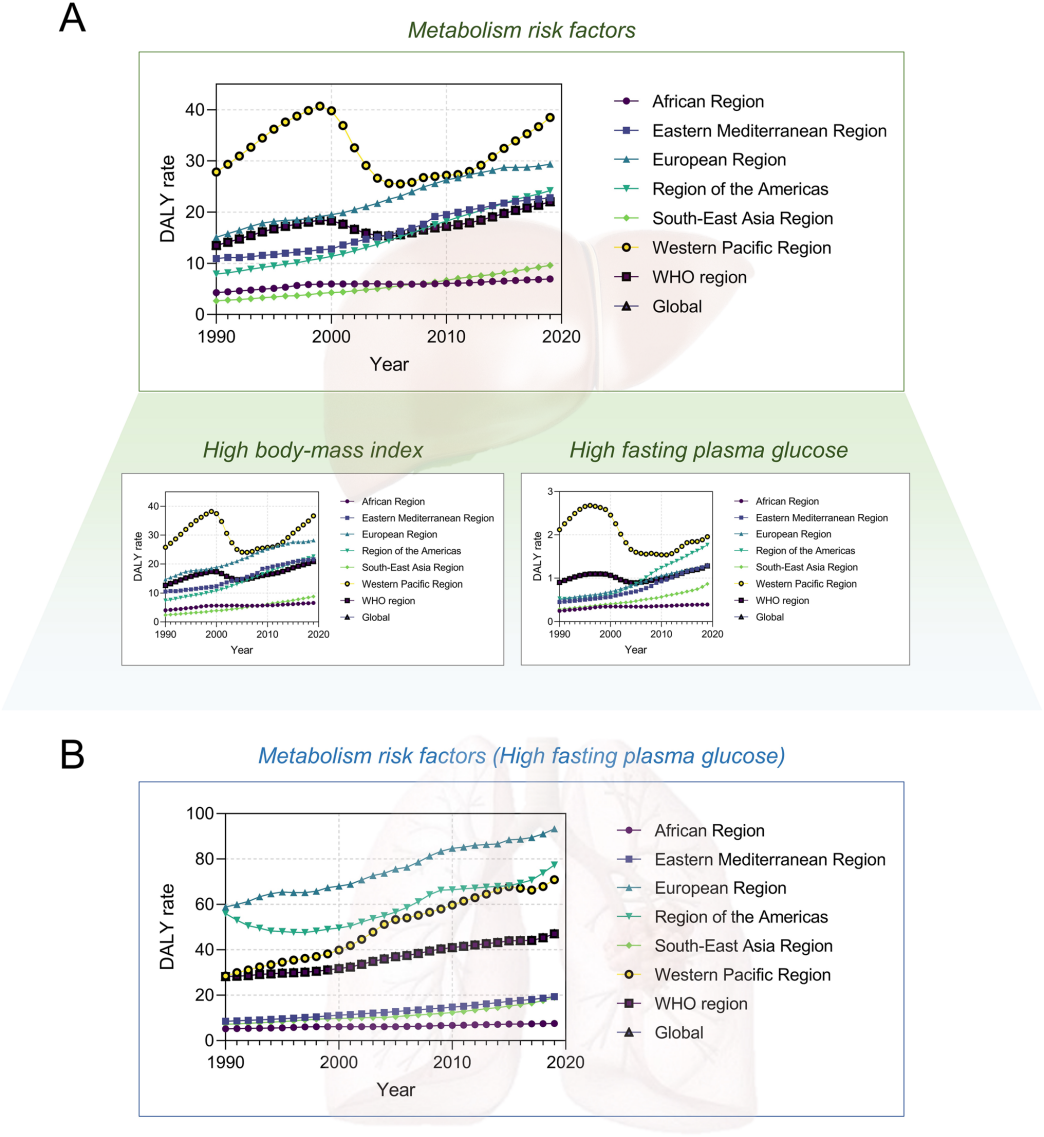
The globalization of the cancer risk factors (metabolic factors). The DALY rate (per 100,000 population) of liver cancer (**A**) and TBL cancers (**B**) from 1990 to 2019 by metabolism risk factors

**Fig. 11:**
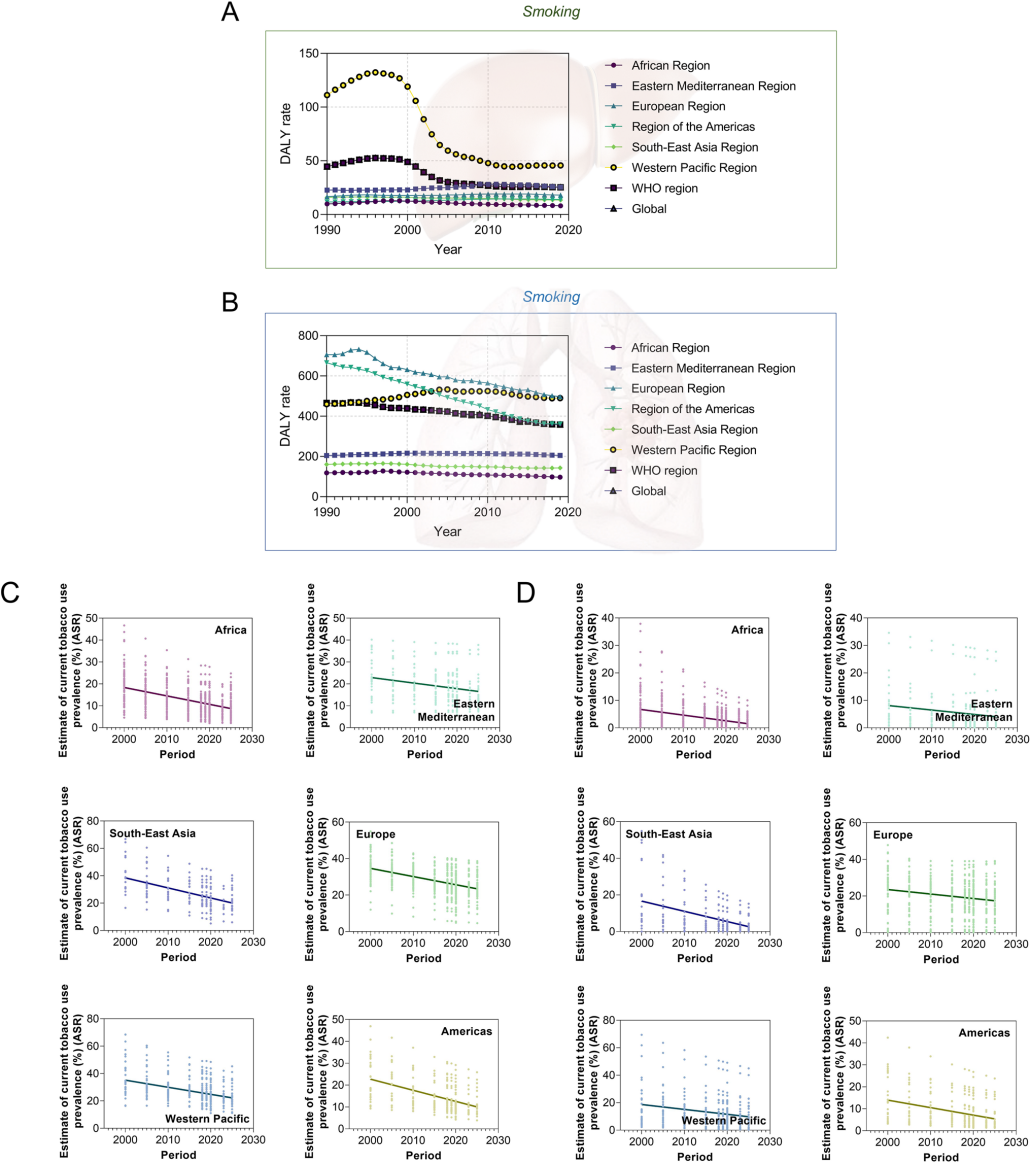
The globalization of the cancer risk factors (smoking). The DALY rate (per 100,000 population) of liver cancer (**A**) and TBL cancers (**B**) from 1990 to 2019 due to smoking. (**C**) The estimated and predicted age-standardized rate (ASR) of tobacco use prevalence in six WHO regions (both sexes) according to WHO. Linear regression best-fit slopes (point estimations with 95% CI): AFR, − 0.3849 [− 0.4389, − 0.3308]; AMR, − 0.5076 [− 0.5891, − 0.4260]; EMR, − 0.2533 [− 0.3749, − 0.1318]; EUR, − 0.4486 [− 0.5076, − 0.3896]; SEAR, − 0.7342 [− 0.9209, − 0.5476]; WPR, − 0.5174 [− 0.6276, − 0.4072]. **D** The estimated and predicted ASR of tobacco use prevalence in six WHO regions (female) according to WHO. Linear regression best-fit slopes (point estimations with 95% CI): AFR, − 0.2079 [− 0.2435, − 0.1723]; AMR, − 0.3419 [− 0.4334, − 0.2505]; EMR, − 0.1616 [− 0.2674, − 0.05575]; EUR, − 0.2452 [− 0.3278, − 0.1625]; SEAR, − 0.5588 [− 0.7306, − 0.3870]; WPR, − 0.3574 [− 0.4923, − 0.2225]

We further explored the globalization of cancer etiology and risk factors over the past decades by investigating cases including the changing etiology of liver cancer, the trends of global HBV vaccination and BMI level, the metabolic risk factors of liver and TBL cancers, and the global trend of smoking prevalence. In general, the changing trends in metabolism risk factors-related DALY rates for both liver and TBL cancer were similar in recent years. The age-standardized incidence (ASI) of liver cancer due to NASH, which is a metabolic disease, also showed similar increasing trends in recent years among all six WHO regions, while the ASI of liver cancer due to HBV and HCV has been stabilized in recent years (Fig. 8b–d). Additionally, the changing trends of ASI for liver cancer due to alcohol use were more diversified recently (decreased in EUR, AFR, and EMR, while increased in other regions) (Fig. 8a). In Fig. 9, the trends of global homogenization in HBV vaccination and BMI level could be observed, where the variances of 3rd dose HepB vaccination have been decreasing in recent years and the age-standardized BMI levels among WHO regions were showing homogenized increasing trends with slopes ranging from 0.05910 to 0.1037. Notably, there was a slight decrease in HepB 3rd dose vaccination coverage in most WHO regions in 2020 and 2021, which may be associated with the COVID-19 pandemic. The COVID-19 pandemic might have several impacts on vaccination coverage. First, the members of WHO suffer from the challenges posed by COVID-19 since the social distancing rules, quarantine policies, and limited human/medical resources have been exerting negative impact on the immunization plans and resources. Second, there has been a decrease in the number of countries reporting immunization data to the WHO in 2020 and 2021 compared to pre-pandemic years, which might be related to the interference caused by the pandemic. However, there has been a rise in HepB vaccination coverage in 2022, indicating that while it has not yet reached pre-pandemic levels, the impact of the COVID-19 pandemic is reducing, which is in line with the fact that 185 countries reported 2022 data through the annual data collection process in 2023, which is comparable to pre-pandemic levels. These changes suggest that countries are gradually recovering from the interruption caused by COVID-19, and as a global factor, the influence of the COVID-19 pandemic is the shared challenge worldwide. In addition, the reported variances of vaccination coverage have experienced a marginal increase during the pandemic, which is potentially attributed to the varying abilities among regions to address the challenges posed by the COVID-19 pandemic (such as the socioeconomic disparities). The absence of data from certain countries could potentially impact the variance of vaccination coverage. Figure 10 shows DALY rates of liver and TBL cancers attributed to the metabolism risk factors from 1990 to 2019 in WHO regions. An obvious increase of DALY rates due to metabolism risk factors for liver cancer was shown in all six WHO regions since 2005 (Fig. 10a). As for TBL cancer, the DALY rates due to metabolism risk factors increased from 1990 to 2019 for all WHO regions except AMR which showed the decrease of DALY rates before 1996 (Fig. 10b). The DALY rate of liver cancer by smoking was low and stable from 1990 to 2019 in most WHO regions except WPR (Fig. 11a). In the regions with high DALY rate due to smoking, the TBL cancer burdens due to smoking have declined rapidly, while the smoking-related TBL cancer burdens were more stable in the regions with low smoking-related DALY rate (Fig. 11b). We further examined the trends of estimated and predicted ASR in tobacco use prevalence among six WHO regions. The result **(**Fig. 11c) showed that the prevalences of tobacco use in WHO regions have been decreasing during the recent decades. Except for SEAR with a decreasing slope of − 0.7342 [− 0.9209, − 0.5476] and EMR with a slope of − 0.2533 [− 0.3749, − 0.1318], the slopes in other four regions were between [− 0.5174, − 0.3849], showing a homogeneous decreasing pattern. Figure 11d further demonstrates the estimated and predicted tobacco use prevalence ASR in WHO regions, where the slopes in most regions were between [− 0.3574, − 0.2079], except for SEAR with a decreasing slope of − 0.5588 [− 0.7306, − 0.3870].

## Discussion

Currently, liver cancer and lung cancer are two of the major cancer types with high morbidity and mortality causing huge social and economic burdens [1, 34–36]. The DALYs of liver cancer and TBL cancer in 2019 worldwide were 12.48 million and 45.69 million, respectively. Both the ASIRs and ASDRs of liver and TBL cancer had decreased globally from 1990 to 2019, though the incident cases, death cases, and DALYs had increased. These inconsistencies between absolute numbers and ASRs may come from the growth of the population worldwide and the aging of the world's population [37].

Different from most of the studies about GBD database which were based on GBD regions [7, 35, 38], our studies about liver and lung cancers focused on the WHO regions. The burden and trends of liver or TBL cancer varied among different WHO regions and even can be totally different. Therefore, it is meaningful to understand the burdens of liver and lung cancer in different WHO regions. Generally, the highest ASIR and ASDR in 2019 for both liver and TBL cancer were in WPR, while the lowest ASIR and ASDR were in AMR for liver cancer and AFR for TBL cancer. As for the DALYs, WPR was the region with the highest DALYs for liver and TBL cancers in 2019 due to its large population, as well as high ASIR and ASDR.

The comparison between liver cancer and lung cancer is meaningful due to the similarities and differences between liver and lung cancer. The main risk factors of both cancers are relatively clear and controllable. For liver cancer, hepatitis virus infection is still the major cause at present causing about 70% of liver cancer deaths [36, 39, 40]. For lung cancer, smoking fully deserves the leading cause with 90% of lung cancer deaths [41]. The control of hepatitis virus and smoking has been proposed for many years and has already made remarkable progress [30, 42–44]. To what extent the control of these major risk factors influences the epidemiology of liver and lung cancer is intriguing and important for policy maintenance or adjustment.

Our results showed that the decrement of ASDR and ASDALY in lung cancer was larger than ASIR partially due to the advances in cancer treatment [45]. The ASIR of lung cancer was stable from 1990 to 2019 globally with a slight decrease, meaning the control of tobacco has worked but at a slower speed. For example, the prevalence of smoking in males is estimated to decrease < 2% from 2010 (33.1%) to 2025 (31.2%) in EMR [46]. Therefore, stronger and durative tobacco control is needed to further extend the decline in lung cancer incidence and it was projected that the ASDR of lung cancer could decrease by 79% by 2065 with a proper decrease in smoking [47]. In order to control cigarettes better, many measures can be taken worldwide and regional-specific. Smoking among youth was remarkable in Africa and South-East Asia countries, as well as other countries [50]. The exposure to cigarette for youth is prominent by the traditional media, such as outdoor TV/movies, billboards, newspapers, and magazines [48, 49, 51, 52]. Therefore, reducing exposure to cigarette (such as reducing or banning cigarette advertising/promotion/sponsorship/movie smoking) are important for youth [53]. Reducing the access to tobacco in youth is also important for tobacco control for exceeding 50% of 13–15 years smokers purchasing cigarettes from the retail store in about half of the countries [54]. In addition, smoke-free policies are also important for these non-smokers due to not only the direct harm of secondhand smoking but also the higher prevalence of susceptibility to initiating smoking in non-smokers who are exposed to secondhand smoke [55, 56]. Populations with lower socioeconomic status are more likely to smoke while the association between policies and smoking was mainly among higher socioeconomic populations [57, 58]. The association between lower income and higher smoking prevalence was found significant in the Americas (OR 1.54), South East Asia (OR 1.53), Europe (OR 1.45), and Western Pacific (OR 1.32) [59]. Therefore, properly increasing the tobacco-control policies or services targeting these low socioeconomic status people may be more efficient and can reduce smoking inequalities [57, 60–62]. In addition, there are many approaches to control smoking such as increasing cigarette taxes [63]. It is also important to restrict smokeless tobacco, which increased steadily recently and has reduced the efficiency of the anti-tobacco campaign in the SEAR [64].

The ASRs of liver cancer showed a rapid decline from 1996 to 2005 globally attributed to the rapid decline of ASRs in WPR, which was probably because of the improvements in sanitary and medical conditions of the low-income countries in WPR. The control of liver cancer mainly relied on the control of HBV and HCV, both of which have made great progress. HBV could be prevented by vaccines while HCV could be cured by oral administration of antiviral agents [42, 65, 66]. In line with the highest ASRs of liver cancer in our study, WPR has the highest prevalence of HBV compared with other regions, especially in China, South East Asia, and Pacific Islands and Territories [67], which means that a decrease in HBV in the WPR is more likely to affect changes around the world. Decreased Hepatitis B surface antigen (HBsAg) seroprevalence in children < 15 years old has happened in WPR due to the HBV vaccine programs, especially in China, Singapore, South Korea, and Malaysia [68]. The lowest prevalence of HBV was also corresponding with the low DALY in EUR and AMR [69]. Despite the low prevalence, the demographic change, immigration increase, and vaccine cost decrease made the cost–benefit ratios supporting the universal HBV vaccination [70]. The coverage of HBV vaccination has improved worldwide. For example, the coverage of HBV vaccination in EMR has increased from 6% in 1992 to 83% in 2014 [71]. The coverage of three doses of HBV vaccination in SEAR has ranged from 56% in 2011 to 87% in 2015 [72]. However, the coverage rate of the HBV vaccine ranged widely across different WHO regions. The coverage of birth dose and three-dose HBV vaccination was lowest in AFR with 10% and 76% in 2015, while the highest coverage was in WPR and AMR, both of which have more than 70% birth dose vaccination coverage and approximately 90% three-dose HBV childhood vaccination coverage [68]. Therefore, it is important to eliminate the inequality in HBV vaccine. The high-risk population of HBV infection also varied among different regions. In WPR, hemodialysis and HIV patients are the populations with the highest risk [68], whereas the prevalence of HBV was higher in drug users, immigrants, and men who have sex with men in most European countries [73]. The current studies about HBV and HCV testing in Europe mainly focus on drug users and healthcare patients, which indicated that more research is needed on immigrants, prison inmates, or men who have sex with men [74].

The exposures to cancer risk factors are increasing and getting more complicated globally, especially in the less-industrialized nations. Globalization brings increased pathogen flows, information flows, and people flows [75]. These flows increased the convergences of epidemiological features of liver and TBL cancers among different WHO regions. One example is that metabolism risk factors are growing worldwide due to the spread of western diet and lifestyles in globalization. The DALY rate attributed to metabolism risk factors are increasing in all six WHO regions for both liver and TBL cancers in recent years regardless of previous fluctuations.

Compared between the control of liver cancer and lung cancer, we found that the successful control of liver cancer is due to the advances in medicine and sanitation while the control of lung cancer relies on the change of lifestyles (smoking) to a greater degree. For the government, medical and health policies are relatively easy to formulate and implement, while the change in people's lifestyles can only be promoted through propaganda and proposal, which depends more on the initiative of the public and are difficult to implement. It is alarming that the ASIR of liver cancer became stable from 2005 to 2019, even showing an increasing trend recently. This reappearance of ASIR growth may be due to the change of the liver cancer etiology, which is characterized by the rapid growth of liver cancer cases due to NASH and alcohol [39]. NASH and alcohol are both tightly related to the lifestyles of humans meaning the control of liver cancer may also shift into a lifestyle-prominent era in the future. Therefore, though huge progress has been made in liver cancer control, we should attach importance to these emerging risk factors together with the goal of eliminating hepatitis virus [42]. The solutions call for both individual behavioral change and, more significantly, imaginative global, collective action from all parties involved [20].

Most importantly, our results highlighted a trend of global homogenization in cancer burdens [12–14, 27–33]. Taking TBL cancer and liver cancer as examples, we studied the convergences of cancer burden metrics and their risk factors in various populations with different ages and sexes among WHO regions. First, the convergences in burden metrics among WHO regions could be witnessed in decreasing variances of ASIRs, ASDALYs, and ASDRs for TBL and liver cancer among WHO memberships during the past decade. Second, the decreasing variances of incidence rate, DALY rate in various age-groups, and their male-to-female ratios among six WHO regions demonstrated that the global convergences in populations are happening in patients with different ages and sexes. Third, although the patterns of etiological and risk factors are undergoing profound changes in many aspects, the convergences of many emerging metabolic and behaviour-related factors could be seen in WHO regions. These results suggest that we need to be acutely aware of the global homogeneity of the disease burden that accompanies increasing globalization, including the global convergences in population, risk factors, and burden indicators.

Our study systematically compared the cancer burden between liver and TBL cancer based on the WHO regions and globally for the first time. However, there are also some limitations in our study. The causes of liver cancer (due to hepatitis B, hepatitis C, alcohol use, NASH, or other causes) were not analyzed in our research since another research has studied it in detail [39]. In addition, the cancer information of some countries is based on prediction due to the lack of data sources, especially in countries/territories with low SDI values. The heterogeneity of cancer burdens in different countries may partially attribute to the diversity of data sources. The limited medical resources in low SDI countries/territories may influence the screening, diagnosis, and treatment of cancers as well as the efforts of medical and health management, which may exacerbate the inaccuracy of the cancer burden data in these countries/territories [7, 23]. The lack of detailed medical information (such as pathological information, neoplasm staging, and therapy) limits the further analysis of these two cancers. Additional data sources, high-quality data, and accurate cancer information may be helpful to overcome these limitations in the future.

The journal *LANCET* published in 2014 the opinion “Cancer is a global and growing, but not uniform, problem” [76]. As the wave of globalization rages, human lifestyles, consumption and dietary habits, and behaviors are facing significant homogenization, which brings about a globalization of potential risk factors and disease burden, of which cancer is a stark example. Today, it is time to rethink and reassess this idea, or, at least, we should face up to the fact that the global homogenization of tumor burden and its risk factors has become a potential trend, which harbors unprecedented opportunities for disease control and should be a phenomenon worthy of attention in public health.

## Conclusions

In summary, we compared the incidence, death, and DALYs of lung and liver cancer based on WHO regions systematically and found the inequities among different regions, sexes, and age groups, which was meaningful for guiding further policy making and resources allocating in the future. There are some interesting findings in this study. First, as for the ASIR and ASDR, the WPR has the most serious burdens for both liver and TBL cancers among six WHO regions. Second, DALYs for both liver and TBL cancer among different WHO regions elevated with the increasing SDI level, except for liver cancer in WPR, which decreased during 1998–2011 and kept increasing since 2011, and TBL cancer in EUR, which showed decrement during 1994–1998 and remained relatively stable since 1998. Third, the analysis of EAPC values indicated that AMR showed the biggest upward trends of liver cancer ASRs, as well as the biggest downward trends of TBL cancer ASRs, followed by EMR. On the contrary, the biggest downward trends of liver cancer ASRs and the biggest upward trends of TBL cancer ASRs were found in WPR. Fourth, according to our results, the importance of lifestyle changes should be emphasized for both lung and liver cancer, especially for liver cancer due to the emerging new risk factors (such as non-alcoholic steatohepatitis and alcohol consumption). Alcohol use was the leading cause of liver cancer death in AFR, EUR, AMR, and SEAR. However, the leading cause of liver cancer death in EMR was high body-mass index, and in WPR was smoking. Smoking was the leading cause of TBL cancer death rate in all six WHO regions in the past three decades. The last but not the least, our results highlighted a trend of global homogenization in cancer burdens. Taking TBL cancer and liver cancer as examples, we studied the convergences of cancer burden metrics and their risk factors in various populations with different ages and sexes among WHO regions reflected from the decreasing variances of metrics, risk factors, and population characteristics.

Our study reflects the commonality and heterogeneity in the disease burden of liver and lung cancer among the six regions within the WHO membership. The different epidemiological characteristics of liver and lung cancers could be derived from the geographical, demographic, and disease background characteristics of the regions themselves. However, in the context of globalization, the quickly spread of medical approaches to prevent, diagnose, and treat cancers, as well as the convergence of risk factors may also contribute to the converged epidemiology of liver and lung cancers to a great extent. Therefore, on the one hand, more attention should be paid to geographical characteristics, socio-demographic levels, and leading causative factors in the prevention and control of chronic non-communicable diseases such as cancers in the future. On the other hand, communications between different regions are essential and helpful due to the convergences of disease epidemiological characteristics with the growing globalization where more united, connected, and interdependent trends are showing in global human lifestyles, consumer behaviours, and commercial activities than those in past centuries. To improve the quantity and quality of health globalization, our study suggests that future health planning should take into account not only the globalization of the cancer burdens themselves, but also the globalization of the underlying risk or protective factors related to human behavior, health economic policies (such as immunization coverage), etc.

### Supplementary Information

Below is the link to the electronic supplementary material.

Supplementary file1 (DOCX 325839 KB)

## Data Availability

The datasets analysed during the current study are available in the GBD 2019 results repository (https://vizhub.healthdata.org/gbd-results), the WHO immunization data, (http://immunizationdata.who.int/pages/coverage/hepb.html), and the Global Health Observatory (GHO) data repository (https://www.who.int/data/gho/data/indicators/indicator-details/GHO).

## Abbreviations

AFP: Alpha-fetoprotein
AFR: African Region (a WHO region)
AMR: Region of the Americas (a WHO region)
ASDR: Age-standardized death rate
ASI: Age-standardized incidence
ASIR: Age-standardized incidence rate
ASRs: Age-standardized rates
DALY: Disability-adjusted life year
EMR: Eastern Mediterranean Region (a WHO region)
EUR: European Region (a WHO region)
GBD: Global Burden of Disease
HBsAg: Hepatitis B surface antigen
HBV: Hepatitis B virus
HCV: Hepatitis C virus
LDCT: Low-dose computed tomography
NASH: Non-alcoholic steatohepatitis
SDI: Sociodemographic index
SEAR: South-East Asia Region (a WHO region)
TBL: Tracheal, bronchus, and lung
WPR: Western Pacific Region (a WHO region)
WHO: World Health Organization
