# Supplementary material for: Cancer Burden Variations and Convergences in Globalization: A Comparative Study on the Tracheal, Bronchus, and Lung (TBL) and Liver Cancer Burdens Among WHO Regions from 1990 to 2019
Source: J Epidemiol Glob Health. 2023 Aug 28;13(4):696–724. doi: 10.1007/s44197-023-00144-x (PMC10686938; doi:10.1007/s44197-023-00144-x)
Supplement: Supplementary file 1 — Supplementary file1 (DOCX 325839 KB) [file 44197_2023_144_MOESM1_ESM.docx]

**Supplementary materials**

Cancer Burden Variations and Convergences in Globalization: A Comparative Study on the Tracheal, Bronchus, and Lung (TBL) and Liver Cancer Burdens among WHO Regions from 1990 to 2019

Mengwei Zhang^1,2,†^, Weiqiu Jin^1,2,†^, Yu Tian^1,2^, Hongda Zhu^1,2^, Ningyuan Zou^1,2^, Yunxuan Jia^3^, Long Jiang^1,2^, Jia Huang^1,2,*^, Yingjie Hu^1,2,*^, and Qingquan Luo^1,2,*^

1. Shanghai Lung Cancer Center, Shanghai Chest Hospital, Shanghai Jiao Tong University, Shanghai, China.

2. School of Medicine, Shanghai Jiao Tong University, Shanghai, China.

3. Department of Thoracic Surgery, Shanghai Chest Hospital, Shanghai Jiao Tong University, Shanghai, China.

† These authors contributed equally to this work.

*** *Correspondence to:*** Huang Jia, [huangjiadragon@126.com](mailto:huangjiadragon@126.com); Hu Yingjie, [jiejie070@163.com](mailto:jiejie070@163.com); Luo Qingquan, [luoqingquan@hotmail.com](mailto:luoqingquan@hotmail.com);

**Table of Contents**

[**Section 1 Studied locations	3**](#_Toc132831434)

[**Section 2 Selected risk factors	5**](#_Toc132831435)

[**Section 3 The ASIR, ASDR, and DALY of liver cancer and TBL cancer among GBD regions and WHO regions based on SDI.	8**](#_Toc132831436)

[**Section 4 Top thirty countries/territories with the most cancer burdens	10**](#_Toc132831437)

[**Section 5 The incidence, death, and DALY of liver and TBL cancer in 1990 and 2019 by sex in different agegroups in WHO regions	21**](#_Toc132831438)

[*Figure S5-1 African Region*	21](#_Toc132831439)

[*Figure S5-2 Eastern Mediterranean Region*	27](#_Toc132831440)

[*Figure S5-3 European Region*	34](#_Toc132831441)

[*Figure S5-4 Region of the Americas*	40](#_Toc132831442)

[*Figure S5-5 South-East Asia Region*	46](#_Toc132831443)

[*Figure S5-6 Western Pacific Region*	52](#_Toc132831444)

[**Section 6 The death rate and DALY of liver and TBL cancer in 1990 and 2019 by risk factors in WHO regions	59**](#_Toc132831445)

[*Figure S6-1 African Region*	59](#_Toc132831446)

[*Figure S6-2 Eastern Mediterranean Region*	68](#_Toc132831447)

[*Figure S6-3 European Region*	78](#_Toc132831448)

[*Figure S6-4 Region of the Americas*	88](#_Toc132831449)

[*Figure S6-5 South-East Asia Region*	98](#_Toc132831450)

[*Figure S6-6 Western Pacific Region*	108](#_Toc132831451)

## Section 1 Studied locations

(A) GBD regions

| **Location ID** | **Location** |
| --- | --- |
| 120 | Andean Latin America |
| 70 | Australasia |
| 104 | Caribbean |
| 32 | Central Asia |
| 42 | Central Europe |
| 124 | Central Latin America |
| 167 | Central Sub-Saharan Africa |
| 5 | East Asia |
| 56 | Eastern Europe |
| 174 | Eastern Sub-Saharan Africa |
| 65 | High-in2come Asia Pacific |
| 100 | High-income North America |
| 138 | North Africa and Middle East |
| 21 | Oceania |
| 159 | South Asia |
| 9 | Southeast Asia |
| 96 | Southern Latin America |
| 192 | Southern Sub-Saharan Africa |
| 134 | Tropical Latin America |
| 73 | Western Europe |
| 199 | Western Sub-Saharan Africa |

(B) WHO regions

| **Location ID** | **Location** |
| --- | --- |
| 479 | WHO region |
| 44563 | African Region |
| 44567 | Eastern Mediterranean Region |
| 44566 | European Region |
| 44564 | Region of the Americas |
| 44565 | South-East Asia Region |
| 44568 | Western Pacific Region |

(C) WHO regional groupings

| **WHO regions** | **Groups** |
| --- | --- |
| **WHO African Region** | Algeria, Angola, Benin, Botswana, Burkina Faso, Burundi, Cabo Verde, Cameroon, Central African Republic, Chad, Comoros, Congo, Côte d’Ivoire, Democratic Republic of the Congo, Equatorial Guinea, Eritrea, Eswatini, Ethiopia, Gabon, Gambia, Ghana, Guinea, Guinea-Bissau, Kenya, Lesotho, Liberia, Madagascar, Malawi, Mali, Mauritania, Mauritius, Mozambique, Namibia, Niger, Nigeria, Rwanda, Sao Tome and Principe, Senegal, Seychelles, Sierra Leone, South Africa, South Sudan, Togo, Uganda, United Republic of Tanzania, Zambia, Zimbabwe |
| **WHO Region of the Americas** | Antigua and Barbuda, Argentina, Bahamas, Barbados, Belize, Bolivia (Plurinational State of), Brazil, Canada, Chile, Colombia, Costa Rica, Cuba, Dominica, Dominican Republic, Ecuador, El Salvador, Grenada, Guatemala, Guyana, Haiti, Honduras, Jamaica, Mexico, Nicaragua, Panama, Paraguay, Peru, Saint Kitts and Nevis, Saint Lucia, Saint Vincent and the Grenadines, Suriname, Trinidad and Tobago, the United States of America, Uruguay, Venezuela (Bolivarian Republic of) |
| **WHO South-East Asia Region** | Bangladesh, Bhutan, Democratic People’s Republic of Korea, India, Indonesia, Maldives, Myanmar, Nepal, Sri Lanka, Thailand, Timor-Leste |
| **WHO European Region** | Albania, Andorra, Armenia, Austria, Azerbaijan, Belarus, Belgium, Bosnia and Herzegovina, Bulgaria, Croatia, Cyprus, Czechia, Denmark, Estonia, Finland, France, Georgia, Germany, Greece, Hungary, Iceland, Ireland, Israel, Italy, Kazakhstan, Kyrgyzstan, Latvia, Lithuania, Luxembourg, Malta, Monaco, Montenegro, Netherlands, North Macedonia, Norway, Poland, Portugal, Republic of Moldova, Romania, Russian Federation, San Marino, Serbia, Slovakia, Slovenia, Spain, Sweden, Switzerland, Tajikistan, Turkey, Turkmenistan, Ukraine, United Kingdom of Great Britain and Northern Ireland, Uzbekistan |
| **WHO Eastern Mediterranean Region** | Afghanistan, Bahrain, Djibouti, Egypt, Iran (Islamic Republic of), Iraq, Jordan, Kuwait, Lebanon, Libya, Morocco, Oman, Pakistan, Qatar, Saudi Arabia, Somalia, Sudan, Syrian Arab Republic, Tunisia, United Arab Emirates, Yemen |
| **WHO Western Pacific Region** | Australia, Brunei Darussalam, Cambodia, China, Cook Islands, Fiji, Japan, Kiribati, Lao People’s Democratic Republic, Malaysia, Marshall Islands, Micronesia (Federated States of), Mongolia, Nauru, New Zealand, Niue, Palau, Papua New Guinea, Philippines, Republic of Korea, Samoa, Singapore, Solomon Islands, Tonga, Tuvalu, Vanuatu, Viet Nam |

## Section 2 Selected risk factors

(A) Liver cancer

| **Risk ID** | **Risk name** |
| --- | --- |
| 118 | Diet high in sugar-sweetened beverages |
| 119 | Diet low in fiber |
| 121 | Diet low in seafood omega-3 fatty acids |
| 122 | Diet low in polyunsaturated fatty acids |
| 123 | Diet high in trans fatty acids |
| 124 | Diet high in sodium |
| 95 | Iron deficiency |
| 96 | Vitamin A deficiency |
| 97 | Zinc deficiency |
| 99 | Smoking |
| 100 | Secondhand smoke |
| 102 | Alcohol use |
| 103 | Drug use |
| 105 | High fasting plasma glucose |
| 107 | High systolic blood pressure |
| 108 | High body-mass index |
| 109 | Low bone mineral density |
| 111 | Diet low in fruits |
| 112 | Diet low in vegetables |
| 113 | Diet low in whole grains |
| 114 | Diet low in nuts and seeds |
| 115 | Diet low in milk |
| 116 | Diet high in red meat |
| 117 | Diet high in processed meat |
| 147 | Diet low in calcium |
| 150 | Occupational exposure to asbestos |
| 151 | Occupational exposure to arsenic |
| 152 | Occupational exposure to benzene |
| 153 | Occupational exposure to beryllium |
| 154 | Occupational exposure to cadmium |
| 155 | Occupational exposure to chromium |
| 156 | Occupational exposure to diesel engine exhaust |
| 158 | Occupational exposure to formaldehyde |
| 159 | Occupational exposure to nickel |
| 160 | Occupational exposure to polycyclic aromatic hydrocarbons |
| 161 | Occupational exposure to silica |
| 162 | Occupational exposure to sulfuric acid |
| 170 | Unsafe sex |
| 237 | Occupational exposure to trichloroethylene |
| 125 | Low physical activity |
| 128 | Occupational asthmagens |
| 129 | Occupational particulate matter, gases, and fumes |
| 130 | Occupational noise |
| 131 | Occupational injuries |
| 132 | Occupational ergonomic factors |
| 134 | Childhood sexual abuse |
| 135 | Intimate partner violence |
| 136 | Non-exclusive breastfeeding |
| 137 | Discontinued breastfeeding |
| 84 | Unsafe sanitation |
| 86 | Ambient particulate matter pollution |
| 87 | Household air pollution from solid fuels |
| 88 | Ambient ozone pollution |
| 90 | Residential radon |
| 91 | Lead exposure |
| 94 | Child underweight |
| 238 | No access to handwashing facility |
| 240 | Child wasting |
| 241 | Child stunting |
| 83 | Unsafe water source |
| 332 | Chewing tobacco |
| 333 | Diet low in legumes |
| 334 | Short gestation |
| 335 | Low birth weight |
| 337 | High temperature |
| 338 | Low temperature |
| 341 | Kidney dysfunction |
| 363 | Bullying victimization |
| 367 | High LDL cholesterol |

(B) TBL cancer

| **Risk ID** | **Risk name** |
| --- | --- |
| 118 | Diet high in sugar-sweetened beverages |
| 119 | Diet low in fiber |
| 121 | Diet low in seafood omega-3 fatty acids |
| 122 | Diet low in polyunsaturated fatty acids |
| 123 | Diet high in trans fatty acids |
| 124 | Diet high in sodium |
| 125 | Low physical activity |
| 127 | Occupational carcinogens |
| 134 | Childhood sexual abuse |
| 135 | Intimate partner violence |
| 147 | Diet low in calcium |
| 95 | Iron deficiency |
| 96 | Vitamin A deficiency |
| 97 | Zinc deficiency |
| 99 | Smoking |
| 100 | Secondhand smoke |
| 170 | Unsafe sex |
| 102 | Alcohol use |
| 103 | Drug use |
| 105 | High fasting plasma glucose |
| 107 | High systolic blood pressure |
| 108 | High body-mass index |
| 109 | Low bone mineral density |
| 111 | Diet low in fruits |
| 112 | Diet low in vegetables |
| 113 | Diet low in whole grains |
| 114 | Diet low in nuts and seeds |
| 115 | Diet low in milk |
| 116 | Diet high in red meat |
| 117 | Diet high in processed meat |
| 82 | Unsafe water, sanitation, and handwashing |
| 88 | Ambient ozone pollution |
| 90 | Residential radon |
| 91 | Lead exposure |
| 93 | Suboptimal breastfeeding |
| 239 | Child growth failure |
| 380 | Particulate matter pollution |
| 332 | Chewing tobacco |
| 333 | Diet low in legumes |
| 337 | High temperature |
| 338 | Low temperature |
| 339 | Low birth weight and short gestation |
| 341 | Kidney dysfunction |
| 363 | Bullying victimization |
| 367 | High LDL cholesterol |

## Section 3 The ASIR, ASDR, and DALY of liver cancer and TBL cancer among GBD regions and WHO regions based on SDI.

**A**

**B**

**C**

**D**

**E**

**F**

**Figure S2** The ASIR, ASDR, and DALY of liver cancer and TBL cancer among GBD regions and WHO regions based on SDI. **A** ASIR of liver cancer. **B** ASIR of TBL cancer. **C** ASDR of liver cancer. **D** ASDR of TBL cancer. **E** DALY of liver cancer. **F** DALY of TBL cancer.

## Section 4 Top thirty countries/territories with the most cancer burdens

**Table S4-1**. Top 30 countries with the highest ASIR metrices in 1990 (Liver cancer)

| **Location** | **1990 ASIR** | **Change** |
| --- | --- | --- |
| Mongolia | 64.22(51.72-77.51) | 0.64(0.22-1.21) |
| Guinea | 29.91(24.86-35.83) | 0.08(-0.28-0.49) |
| Gambia | 29.75(22.26-39.05) | 0.28(-0.15-0.86) |
| China | 25.71(21.73-30.35) | -0.59(-0.68--0.48) |
| Tonga | 23.33(16.2-30.14) | 0.04(-0.25-0.44) |
| Thailand | 20.81(18.13-23.74) | 0.16(-0.16-0.6) |
| Egypt | 16.75(13.1-20.2) | 0.25(-0.17-0.94) |
| Qatar | 16.14(12.14-21.04) | 0.08(-0.25-0.54) |
| Mali | 15.12(12.43-18.25) | -0.04(-0.31-0.28) |
| Democratic People's Republic of Korea | 14.62(11.35-18.97) | -0.3(-0.51--0.04) |
| Japan | 14.22(13.71-14.62) | -0.11(-0.25-0.05) |
| Kiribati | 13.92(10.8-17.27) | -0.16(-0.4-0.16) |
| Cook Islands | 13.25(11.01-15.61) | -0.14(-0.34-0.1) |
| Saint Kitts and Nevis | 12.71(11.56-14.03) | -0.71(-0.76--0.64) |
| Taiwan | 12.26(11.74-12.77) | -0.29(-0.45--0.09) |
| Zimbabwe | 11.76(9.21-17.91) | 0.16(-0.21-0.6) |
| Marshall Islands | 11.66(8.89-15.25) | -0.12(-0.36-0.2) |
| Afghanistan | 11.48(8.93-14.53) | -0.14(-0.38-0.16) |
| Micronesia | 11.16(8.3-14.48) | -0.07(-0.39-0.35) |
| Honduras | 11.06(4.16-16.11) | 0.34(0.02-0.95) |
| Republic of Korea | 10.99(9.18-13.42) | 1.07(0.57-1.74) |
| Cambodia | 10.86(8.82-13.11) | -0.15(-0.38-0.19) |
| Albania | 10.85(10.1-11.6) | -0.39(-0.56--0.19) |
| Nauru | 10.54(7.58-13.38) | -0.13(-0.36-0.19) |
| Tuvalu | 10.4(7.88-13.47) | -0.19(-0.41-0.15) |
| Lao People's Democratic Republic | 9.99(6.95-13.51) | -0.33(-0.55--0.01) |
| Brunei Darussalam | 9.68(7.88-11.49) | 0.21(-0.03-0.53) |
| Philippines | 9.64(7.27-11.92) | -0.34(-0.52--0.04) |
| Palau | 9.64(6.66-13.3) | 0.03(-0.31-0.57) |
| Andorra | 9.57(7.16-12.99) | 0.14(-0.23-0.67) |

**Table S4-2**. Top 30 countries with the highest ASIR metrices in 2019 (Liver cancer)

| **Location** | **2019 ASIR** | **Change** |
| --- | --- | --- |
| Mongolia | 105.22(82.57-131.46) | 0.64(0.22-1.21) |
| Gambia | 38.21(27.53-49.67) | 0.28(-0.15-0.86) |
| Guinea | 32.17(22.33-41.9) | 0.08(-0.28-0.49) |
| Tonga | 24.33(17.65-31.9) | 0.04(-0.25-0.44) |
| Thailand | 24.18(17.89-32.01) | 0.16(-0.16-0.6) |
| Republic of Korea | 22.8(18.72-27.32) | 1.07(0.57-1.74) |
| Egypt | 20.92(15.09-28.51) | 0.25(-0.17-0.94) |
| Eswatini | 18.43(5.61-32.97) | 2.27(-0.4-6.71) |
| Qatar | 17.39(12.83-22.91) | 0.08(-0.25-0.54) |
| Honduras | 14.8(6.8-21.7) | 0.34(0.02-0.95) |
| Mali | 14.46(10.75-18.98) | -0.04(-0.31-0.28) |
| Lesotho | 14.44(6.05-22.7) | 1.81(-0.29-4.98) |
| Zimbabwe | 13.64(10.27-18.13) | 0.16(-0.21-0.6) |
| Japan | 12.71(10.51-14.98) | -0.11(-0.25-0.05) |
| Brunei Darussalam | 11.75(9.96-13.78) | 0.21(-0.03-0.53) |
| Kiribati | 11.69(9.09-14.66) | -0.16(-0.4-0.16) |
| Cabo Verde | 11.56(9.52-13.94) | 9.58(7.4-12.48) |
| Singapore | 11.5(9.24-14.41) | 0.36(0.1-0.72) |
| Cook Islands | 11.37(9.15-13.93) | -0.14(-0.34-0.1) |
| Andorra | 10.95(8.23-14.37) | 0.14(-0.23-0.67) |
| China | 10.46(8.74-12.42) | -0.59(-0.68--0.48) |
| Micronesia | 10.37(6.93-14.51) | -0.07(-0.39-0.35) |
| Democratic People's Republic of Korea | 10.23(7.66-13.36) | -0.3(-0.51--0.04) |
| Marshall Islands | 10.22(7.38-13.84) | -0.12(-0.36-0.2) |
| Palau | 9.97(7.68-12.94) | 0.03(-0.31-0.57) |
| Afghanistan | 9.82(7.63-12.44) | -0.14(-0.38-0.16) |
| Cambodia | 9.2(7.26-11.26) | -0.15(-0.38-0.19) |
| Nauru | 9.17(6.5-12.59) | -0.13(-0.36-0.19) |
| Vanuatu | 9.05(6.21-12.85) | -0.02(-0.33-0.45) |
| Monaco | 9.04(7.13-11.28) | 1.61(0.93-2.55) |

**Table S4-3**. Top 30 countries with the highest ASDR metrices in 1990 (Liver cancer)

| **Location** | **1990 ASDR** | **Change** |
| --- | --- | --- |
| Mongolia | 66.77(54.18-79.9) | -0.64(-0.71--0.53) |
| Guinea | 31.8(26.71-38.1) | -0.31(-0.52--0.06) |
| Gambia | 30.76(23.19-40.07) | -0.39(-0.53--0.22) |
| China | 25.99(22.29-30.55) | -0.15(-0.38-0.19) |
| Tonga | 23.93(16.74-30.82) | -0.07(-0.25-0.16) |
| Thailand | 21.6(18.83-24.52) | -0.32(-0.54--0.02) |
| Egypt | 17.43(13.67-20.93) | 0.08(-0.22-0.45) |
| Qatar | 17.37(12.99-22.54) | -0.23(-0.49-0.15) |
| Mali | 15.7(12.98-18.83) | 0.46(0.01-1.02) |
| Democratic People's Republic of Korea | 14.77(11.51-19.02) | -0.35(-0.52--0.05) |
| Kiribati | 14.29(11.14-17.77) | 0.28(-0.11-0.74) |
| Cook Islands | 13.6(11.34-15.98) | 0.11(-0.19-0.52) |
| Saint Kitts and Nevis | 13.47(12.28-14.87) | -0.16(-0.41-0.18) |
| Zimbabwe | 12.16(9.51-18.38) | -0.31(-0.52-0.05) |
| Marshall Islands | 12.14(9.19-15.86) | 0.05(-0.24-0.49) |
| Taiwan | 11.98(11.46-12.47) | -0.16(-0.4-0.15) |
| Afghanistan | 11.97(9.38-15.15) | -0.13(-0.36-0.19) |
| Honduras | 11.92(4.4-17.38) | -0.08(-0.38-0.33) |
| Japan | 11.67(11.25-11.96) | 0.1(-0.2-0.48) |
| Cambodia | 11.62(9.34-13.98) | -0.18(-0.4-0.08) |
| Albania | 11.62(10.81-12.44) | -0.19(-0.41-0.14) |
| Micronesia | 11.58(8.62-14.99) | 0.03(-0.25-0.42) |
| Republic of Korea | 11.04(9.25-13.4) | -0.03(-0.33-0.43) |
| Nauru | 10.89(7.88-13.83) | 5.26(4.17-6.46) |
| Tuvalu | 10.76(8.16-13.96) | 3.28(2.16-5.13) |
| Lao People's Democratic Republic | 10.47(7.33-14.06) | 2.03(1.46-2.75) |
| Brunei Darussalam | 10.13(8.33-12.08) | 0.83(0.56-1.13) |
| Guatemala | 10.05(8.95-11.17) | 1.05(0.72-1.41) |
| Philippines | 10.04(7.53-12.27) | 0.73(0.3-1.3) |
| Palau | 9.78(6.71-13.43) | 2.77(1.97-3.73) |

**Table S4-4**. Top 30 countries with the highest ASDR metrices in 2019 (Liver cancer)

| **Location** | **2019 ASDR** | **Change** |
| --- | --- | --- |
| Mongolia | 115.23(91.48-142.48) | 0.73(0.3-1.3) |
| Gambia | 39.51(29.01-50.99) | 0.28(-0.14-0.84) |
| Guinea | 34.05(23.98-44.01) | 0.07(-0.29-0.48) |
| Tonga | 24.74(18.09-32.04) | 0.03(-0.25-0.42) |
| Thailand | 24.01(17.88-31.65) | 0.11(-0.19-0.52) |
| Egypt | 21.25(15.44-28.92) | 0.22(-0.18-0.89) |
| Eswatini | 19.09(5.98-33.88) | 2.21(-0.39-6.45) |
| Republic of Korea | 16.2(14.47-17.94) | 0.47(0.16-0.86) |
| Honduras | 16.14(7.41-23.5) | 0.35(0.04-0.98) |
| Qatar | 15.88(11.76-20.79) | -0.09(-0.37-0.3) |
| Lesotho | 15.17(6.45-23.71) | 1.8(-0.27-4.82) |
| Mali | 15.03(11.25-19.52) | -0.04(-0.3-0.28) |
| Zimbabwe | 14.03(10.58-18.67) | 0.15(-0.22-0.6) |
| Cabo Verde | 12.34(10.2-14.87) | 9.52(7.42-12.43) |
| Kiribati | 11.95(9.29-15.08) | -0.16(-0.4-0.15) |
| Brunei Darussalam | 11.53(9.82-13.38) | 0.14(-0.08-0.43) |
| Cook Islands | 11.13(9.03-13.51) | -0.18(-0.36-0.04) |
| Micronesia | 10.7(7.24-14.74) | -0.08(-0.38-0.33) |
| Marshall Islands | 10.57(7.64-14.31) | -0.13(-0.36-0.19) |
| Afghanistan | 10.27(7.98-12.93) | -0.14(-0.37-0.16) |
| Democratic People's Republic of Korea | 10.2(7.75-13.3) | -0.31(-0.52--0.06) |
| Cambodia | 9.86(7.85-12.03) | -0.15(-0.38-0.19) |
| Palau | 9.79(7.59-12.53) | 0(-0.32-0.5) |
| Andorra | 9.44(7.14-12.24) | 0.01(-0.33-0.45) |
| China | 9.41(7.95-11.13) | -0.64(-0.71--0.53) |
| Nauru | 9.39(6.7-12.73) | -0.14(-0.36-0.16) |
| Vanuatu | 9.36(6.41-13.2) | -0.03(-0.33-0.43) |
| North Macedonia | 8.82(7.01-11.1) | -0.03(-0.25-0.23) |
| Japan | 8.78(7.8-9.42) | -0.25(-0.31--0.21) |
| Tuvalu | 8.72(6.51-11.52) | -0.19(-0.41-0.13) |

**Table S4-5**. Top 30 countries with the highest ASDALY metrices in 1990 (Liver cancer)

| **Location** | **1990 ASDALY** | **Change** |
| --- | --- | --- |
| Mongolia | 1726.2(1371.23-2109.52) | 0.48(0.08-1.04) |
| Gambia | 876.47(636.2-1170.09) | 0.27(-0.15-0.91) |
| Guinea | 833.45(689.56-1014.67) | 0.07(-0.3-0.49) |
| China | 769.11(653.21-913.07) | -0.66(-0.73--0.55) |
| Tonga | 655.95(453.35-861.63) | 0.04(-0.26-0.5) |
| Thailand | 575.32(500.14-659.92) | 0.07(-0.24-0.49) |
| Egypt | 457.68(366.19-548.17) | 0.18(-0.22-0.84) |
| Mali | 445.32(363.38-537.01) | -0.08(-0.35-0.25) |
| Democratic People's Republic of Korea | 437.07(332.88-577.51) | -0.31(-0.55--0.02) |
| Kiribati | 423.71(325.23-531.34) | -0.17(-0.41-0.15) |
| Cambodia | 371.64(291.05-482.64) | -0.23(-0.5-0.1) |
| Qatar | 361.9(274.31-471.5) | -0.21(-0.47-0.16) |
| Cook Islands | 360.57(296.86-431.52) | -0.18(-0.39-0.09) |
| Taiwan | 346.27(332.07-360.69) | -0.49(-0.61--0.34) |
| Saint Kitts and Nevis | 327.88(296.71-362.83) | -0.72(-0.78--0.65) |
| Marshall Islands | 325.73(249.51-422.87) | -0.11(-0.36-0.22) |
| Afghanistan | 324.06(247.55-417.52) | -0.16(-0.39-0.17) |
| Zimbabwe | 317.2(244.83-495.24) | 0.18(-0.21-0.64) |
| Micronesia | 315.59(230.11-415.7) | -0.08(-0.42-0.38) |
| Nauru | 298.17(208.98-392.54) | -0.14(-0.37-0.2) |
| Republic of Korea | 297.39(245.74-363.22) | 0.31(0.03-0.68) |
| Tuvalu | 293.76(220.25-379.35) | -0.19(-0.42-0.16) |
| Japan | 292.18(284.23-298.37) | -0.4(-0.44--0.37) |
| Honduras | 283.36(112.98-410) | 0.27(-0.04-0.82) |
| Lao People's Democratic Republic | 281.66(191.88-392.48) | -0.36(-0.59-0) |
| Palau | 279.31(191.65-391.63) | 0.03(-0.33-0.59) |
| Philippines | 276.14(207.37-336.12) | -0.35(-0.53--0.06) |
| Albania | 271.62(253.28-290.59) | -0.41(-0.57--0.2) |
| Vanuatu | 255.08(164.52-385.91) | 0(-0.33-0.51) |
| Guatemala | 240.23(212.63-268.67) | -0.53(-0.64--0.38) |

**Table S4-6**. Top 30 countries with the highest ASDALY metrices in 2019 (Liver cancer)

| **Location** | **2019 ASDALY** | **Change** |
| --- | --- | --- |
| Mongolia | 2558.12(1961.96-3278.63) | 0.48(0.08-1.04) |
| Gambia | 1116.06(789.18-1480.33) | 0.27(-0.15-0.91) |
| Guinea | 889.94(611.41-1163.89) | 0.07(-0.3-0.49) |
| Tonga | 684.06(487.15-913.98) | 0.04(-0.26-0.5) |
| Thailand | 615.25(450.05-818.04) | 0.07(-0.24-0.49) |
| Egypt | 537.85(382.38-742.42) | 0.18(-0.22-0.84) |
| Eswatini | 534.72(151.32-1000.58) | 2.45(-0.42-7.48) |
| Lesotho | 410.68(162.44-658.81) | 1.91(-0.33-5.45) |
| Mali | 408.97(298.64-547.03) | -0.08(-0.35-0.25) |
| Republic of Korea | 390.81(348.58-435.93) | 0.31(0.03-0.68) |
| Zimbabwe | 373.68(274.31-511.59) | 0.18(-0.21-0.64) |
| Honduras | 359.62(166.22-538.16) | 0.27(-0.04-0.82) |
| Kiribati | 353.67(273.08-446.53) | -0.17(-0.41-0.15) |
| Cabo Verde | 303.59(245.48-372.44) | 9.75(7.43-12.67) |
| Democratic People's Republic of Korea | 299.93(216.26-405.84) | -0.31(-0.55--0.02) |
| Cook Islands | 295.21(230.25-366.11) | -0.18(-0.39-0.09) |
| Micronesia | 290.57(185.95-424.89) | -0.08(-0.42-0.38) |
| Marshall Islands | 288.32(205.15-393.78) | -0.11(-0.36-0.22) |
| Qatar | 287.67(206.42-382.88) | -0.21(-0.47-0.16) |
| Palau | 287.38(216.5-375.81) | 0.03(-0.33-0.59) |
| Cambodia | 284.56(217.91-357.03) | -0.23(-0.5-0.1) |
| Brunei Darussalam | 284.15(241.01-336.53) | 0.19(-0.06-0.53) |
| Afghanistan | 271.95(207.43-351.12) | -0.16(-0.39-0.17) |
| China | 264.31(220.69-315.14) | -0.66(-0.73--0.55) |
| Nauru | 256.69(177.05-363.21) | -0.14(-0.37-0.2) |
| Vanuatu | 255.58(172.13-366.89) | 0(-0.33-0.51) |
| Tuvalu | 238.33(173.39-325.61) | -0.19(-0.42-0.16) |
| Northern Mariana Islands | 210.02(169.68-261.41) | 0.13(-0.17-0.51) |
| Andorra | 207.57(155.78-273.08) | 0(-0.35-0.47) |
| North Macedonia | 202.54(158.3-259.74) | -0.09(-0.31-0.18) |

**Table S4-7**. Top 30 countries with the highest ASIR metrices in 1990 (TBL cancer)

| **Location** | **1990 ASIR** | **Change** |
| --- | --- | --- |
| Greenland | 85.59(77.79-95.54) | -0.09(-0.26-0.08) |
| United States of America | 58.87(57.24-59.96) | -0.23(-0.34--0.11) |
| Northern Mariana Islands | 55.9(48.24-65.55) | -0.2(-0.33--0.05) |
| Denmark | 52.86(51.14-54.47) | -0.19(-0.37-0.02) |
| Canada | 52.15(50.42-53.77) | -0.16(-0.35-0.06) |
| Belgium | 51.59(49.65-53.54) | -0.23(-0.4--0.02) |
| Netherlands | 50.22(48.58-51.69) | -0.11(-0.3-0.12) |
| United Kingdom | 49.95(48.58-50.78) | -0.2(-0.33--0.05) |
| Hungary | 49.27(47.7-50.73) | 0.05(-0.14-0.28) |
| Czechia | 48.77(47.34-50.65) | -0.33(-0.45--0.17) |
| Montenegro | 48.2(42.22-53.95) | 0.18(-0.06-0.48) |
| Monaco | 47.77(38.25-57.9) | 0.58(0.19-1.1) |
| Brunei Darussalam | 46.62(38.32-55.13) | -0.01(-0.21-0.24) |
| Slovakia | 45.85(43.5-48.17) | -0.27(-0.44--0.03) |
| Croatia | 45.27(42.19-48.54) | -0.11(-0.31-0.13) |
| Poland | 45.05(44.24-45.79) | -0.04(-0.2-0.14) |
| Andorra | 45.02(34.92-60.37) | -0.12(-0.39-0.22) |
| Luxembourg | 44.29(41.89-46.94) | -0.27(-0.4--0.1) |
| Uruguay | 43.5(41.56-45.51) | -0.22(-0.4-0) |
| Ukraine | 42.79(41.25-44.19) | -0.36(-0.48--0.23) |
| Singapore | 42.72(40.64-44.53) | -0.34(-0.46--0.18) |
| Greece | 42.58(40.79-44.29) | -0.01(-0.23-0.25) |
| Kazakhstan | 42.5(40.43-44.73) | -0.5(-0.57--0.42) |
| Palau | 42.5(33.91-53.83) | 0.05(-0.23-0.37) |
| Bahrain | 41.42(35.26-47.78) | -0.52(-0.65--0.35) |
| Mongolia | 40.32(33.4-47.7) | -0.23(-0.41-0.03) |
| Switzerland | 40.04(38.55-41.46) | -0.35(-0.48--0.17) |
| Estonia | 39.89(38.49-41.41) | -0.29(-0.45--0.11) |
| Nauru | 39.68(27.04-54.28) | -0.03(-0.27-0.28) |
| Ireland | 39.68(38.04-41.2) | -0.16(-0.33-0.07) |

**Table S4-8**. Top 30 countries with the highest ASIR metrices in 2019 (TBL cancer)

| **Location** | **2019 ASIR** | **Change** |
| --- | --- | --- |
| Greenland | 77.71(64.38-90.6) | -0.09(-0.26-0.08) |
| Monaco | 75.57(61.39-90.82) | 0.58(0.19-1.1) |
| Montenegro | 56.72(46.5-68.94) | 0.18(-0.06-0.48) |
| Hungary | 51.94(42.61-63.65) | 0.05(-0.14-0.28) |
| Serbia | 49.38(38.82-62.4) | 0.26(-0.03-0.66) |
| Brunei Darussalam | 46.15(40.91-51.83) | -0.01(-0.21-0.24) |
| United States of America | 45.13(39.11-52.21) | -0.23(-0.34--0.11) |
| Netherlands | 44.82(35.35-56.6) | -0.11(-0.3-0.12) |
| Northern Mariana Islands | 44.82(38.99-50.05) | -0.2(-0.33--0.05) |
| Palau | 44.54(36.03-55.03) | 0.05(-0.23-0.37) |
| Canada | 43.63(33.99-55.05) | -0.16(-0.35-0.06) |
| Poland | 43.06(35.98-51.42) | -0.04(-0.2-0.14) |
| Denmark | 42.91(33.7-53.14) | -0.19(-0.37-0.02) |
| Greece | 41.98(32.69-53.06) | -0.01(-0.23-0.25) |
| China | 41.71(35.22-48.8) | 0.38(0.11-0.74) |
| North Macedonia | 40.42(31.04-51.24) | 0.37(0.05-0.76) |
| Croatia | 40.39(31.62-51.05) | -0.11(-0.31-0.13) |
| Bosnia and Herzegovina | 40.26(31.35-50.7) | 0.06(-0.18-0.34) |
| United Kingdom | 40.16(33.54-47.33) | -0.2(-0.33--0.05) |
| Belgium | 39.84(31.05-50.57) | -0.23(-0.4--0.02) |
| Andorra | 39.49(30.5-49.93) | -0.12(-0.39-0.22) |
| Nauru | 38.45(25.3-50.66) | -0.03(-0.27-0.28) |
| San Marino | 36.85(27.73-48.61) | 0.01(-0.26-0.38) |
| France | 36.82(28.64-46.99) | 0.09(-0.16-0.39) |
| Bulgaria | 36.36(28.73-45.51) | 0.27(0-0.61) |
| Cuba | 35.88(29.37-43.68) | 0.08(-0.12-0.3) |
| Republic of Korea | 35.61(29.58-41.94) | 0.45(0.21-0.74) |
| Germany | 35.48(27.82-46.13) | 0.05(-0.18-0.36) |
| Iceland | 34.55(30.17-39.47) | -0.05(-0.19-0.1) |
| Uruguay | 34.13(26.64-42.94) | -0.22(-0.4-0) |

**Table S4-9**. Top 30 countries with the highest ASDR metrices in 1990 (TBL cancer)

| **Location** | **1990 ASDR** | **Change** |
| --- | --- | --- |
| Greenland | 87.78(79.76-97.23) | -0.11(-0.28-0.07) |
| Northern Mariana Islands | 58.72(50.72-68.68) | -0.21(-0.34--0.07) |
| Belgium | 50.2(48.43-51.89) | -0.3(-0.34--0.26) |
| Denmark | 49.89(48.36-51.26) | -0.26(-0.32--0.2) |
| United States of America | 49.35(47.82-50.29) | -0.27(-0.3--0.24) |
| Brunei Darussalam | 47.79(39.36-56.55) | -0.06(-0.25-0.17) |
| Hungary | 47.73(46.29-49.08) | 0.01(-0.17-0.22) |
| Netherlands | 47.47(46.06-48.78) | -0.2(-0.24--0.15) |
| Czechia | 46.91(45.6-48.57) | -0.38(-0.49--0.23) |
| United Kingdom | 46.34(45.05-47.1) | -0.29(-0.32--0.26) |
| Canada | 46.19(44.65-47.35) | -0.26(-0.3--0.22) |
| Montenegro | 46.07(40.39-51.47) | 0.16(-0.07-0.45) |
| Bahrain | 46.06(39.19-53.05) | -0.52(-0.65--0.35) |
| Poland | 45.88(44.97-46.67) | -0.03(-0.2-0.15) |
| Palau | 44.68(35.83-56.51) | 0.02(-0.24-0.33) |
| Monaco | 44.02(35.47-53.13) | 0.46(0.12-0.92) |
| Uruguay | 43.27(41.55-45.03) | -0.23(-0.28--0.17) |
| Andorra | 43.1(33.61-57.25) | -0.21(-0.45-0.1) |
| Luxembourg | 42.94(40.7-45.11) | -0.32(-0.41--0.23) |
| Mongolia | 42.61(35.36-50.24) | -0.22(-0.4-0.04) |
| Nauru | 41.94(28.92-57.14) | -0.04(-0.28-0.26) |
| Kazakhstan | 41.86(39.78-44.04) | -0.49(-0.57--0.42) |
| Croatia | 41.44(38.64-44.32) | -0.19(-0.37-0.02) |
| Greece | 41.35(39.71-42.85) | -0.09(-0.15--0.03) |
| Guam | 40.33(33.99-47.23) | -0.21(-0.37-0.01) |
| Singapore | 39.91(38.14-41.47) | -0.48(-0.52--0.44) |
| Estonia | 39.68(38.3-41.17) | -0.31(-0.46--0.14) |
| Slovakia | 39.49(37.48-41.43) | -0.32(-0.48--0.1) |
| Bermuda | 39.32(36.68-42.24) | -0.39(-0.48--0.26) |
| Turkey | 39.25(31.21-48.16) | -0.14(-0.39-0.18) |

**Table S4-10**. Top 30 countries with the highest ASDR metrices in 2019 (TBL cancer)

| **Location** | **2019 ASDR** | **Change** |
| --- | --- | --- |
| Greenland | 78.23(63.91-91.97) | -0.11(-0.28-0.07) |
| Monaco | 64.23(52.32-76.9) | 0.46(0.12-0.92) |
| Montenegro | 53.36(43.82-64.43) | 0.16(-0.07-0.45) |
| Hungary | 48.12(39.58-58.44) | 0.01(-0.17-0.22) |
| Northern Mariana Islands | 46.24(40.35-51.51) | -0.21(-0.34--0.07) |
| Serbia | 45.96(36.13-57.51) | 0.19(-0.08-0.57) |
| Palau | 45.79(37.28-56.29) | 0.02(-0.24-0.33) |
| Brunei Darussalam | 44.83(39.66-50.19) | -0.06(-0.25-0.17) |
| Poland | 44.31(36.96-52.61) | -0.03(-0.2-0.15) |
| Nauru | 40.44(27.06-52.79) | -0.04(-0.28-0.26) |
| Bosnia and Herzegovina | 39.44(30.89-49.42) | 0.04(-0.2-0.3) |
| North Macedonia | 38.89(30.04-49.11) | 0.33(0.03-0.71) |
| China | 38.7(32.8-45.03) | 0.24(0-0.54) |
| Netherlands | 38.1(35.37-40.47) | -0.2(-0.24--0.15) |
| Greece | 37.6(35.3-39.76) | -0.09(-0.15--0.03) |
| Denmark | 37(34.15-40.02) | -0.26(-0.32--0.2) |
| United States of America | 36.11(34.09-37.51) | -0.27(-0.3--0.24) |
| Belgium | 35.12(32.93-37.14) | -0.3(-0.34--0.26) |
| Cuba | 34.19(28.1-41.53) | 0(-0.18-0.21) |
| Andorra | 34.18(26.49-42.94) | -0.21(-0.45-0.1) |
| Solomon Islands | 34.14(19.83-53.02) | 0.12(-0.19-0.48) |
| Canada | 34.09(31.42-36.13) | -0.26(-0.3--0.22) |
| Bulgaria | 34.03(26.93-42.61) | 0.23(-0.03-0.55) |
| Turkey | 33.77(26.89-41.8) | -0.14(-0.39-0.18) |
| Uruguay | 33.46(31.29-35.5) | -0.23(-0.28--0.17) |
| Croatia | 33.38(26.32-42.13) | -0.19(-0.37-0.02) |
| Mongolia | 33.35(26.47-41.97) | -0.22(-0.4-0.04) |
| Micronesia | 32.95(20.51-47.2) | 0.13(-0.23-0.57) |
| United Kingdom | 32.85(30.83-34.33) | -0.29(-0.32--0.26) |
| San Marino | 32.52(21.24-47.01) | -0.05(-0.39-0.42) |

**Table S4-11**. Top 30 countries with the highest ASDALY metrices in 1990 (TBL cancer)

| **Location** | **1990 ASDALY** | **Change** |
| --- | --- | --- |
| Greenland | 2174.56(1953.04-2432.11) | -0.19(-0.35--0.01) |
| Northern Mariana Islands | 1350.27(1166.03-1609.25) | -0.24(-0.38--0.1) |
| Hungary | 1295.93(1255.74-1333.8) | -0.06(-0.24-0.15) |
| Czechia | 1238.29(1201.82-1284.59) | -0.46(-0.57--0.33) |
| Poland | 1224.74(1205.37-1244.49) | -0.14(-0.29-0.03) |
| Denmark | 1218.32(1184.29-1250.34) | -0.35(-0.4--0.3) |
| Montenegro | 1205.26(1052.86-1353.47) | 0.11(-0.12-0.4) |
| United States of America | 1197.78(1172.11-1216.85) | -0.36(-0.38--0.34) |
| Belgium | 1185.72(1148.32-1224.87) | -0.33(-0.37--0.29) |
| Kazakhstan | 1175.24(1116.63-1238.9) | -0.55(-0.62--0.48) |
| Uruguay | 1121.48(1078.74-1166.57) | -0.28(-0.33--0.22) |
| Netherlands | 1107.03(1078.86-1135.2) | -0.25(-0.3--0.21) |
| Canada | 1104.81(1076.64-1130.67) | -0.35(-0.39--0.31) |
| Ukraine | 1099.2(1061.76-1135.53) | -0.43(-0.53--0.31) |
| Croatia | 1097.02(1020.92-1177.37) | -0.26(-0.44--0.04) |
| Monaco | 1081.09(870.14-1315.53) | 0.37(0.01-0.83) |
| Estonia | 1063.6(1025.4-1105.84) | -0.4(-0.54--0.25) |
| Palau | 1061.91(839.69-1359.19) | 0.01(-0.26-0.34) |
| Slovakia | 1058.77(1001.62-1110.77) | -0.39(-0.54--0.19) |
| Luxembourg | 1051(997.2-1107.35) | -0.38(-0.46--0.29) |
| Serbia | 1044.47(875.55-1145.84) | 0.13(-0.14-0.5) |
| United Kingdom | 1043.53(1022.21-1057.8) | -0.36(-0.38--0.34) |
| Armenia | 1039.47(990.88-1089.88) | -0.26(-0.38--0.11) |
| Nauru | 1019.2(677.33-1417.36) | -0.06(-0.3-0.25) |
| Turkey | 1009.45(782.79-1260.22) | -0.19(-0.43-0.13) |
| Andorra | 997.94(767.74-1345.46) | -0.22(-0.47-0.1) |
| Brunei Darussalam | 990.1(813.99-1175.05) | -0.12(-0.3-0.11) |
| Mongolia | 987.42(806.65-1179.65) | -0.26(-0.45-0) |
| Bosnia and Herzegovina | 984.61(930.3-1039.23) | -0.03(-0.26-0.24) |
| Greece | 960.23(926.75-991.73) | -0.11(-0.16--0.05) |

**Table S4-12**. Top 30 countries with the highest ASDALY metrices in 2019 (TBL cancer)

| **Location** | **2019 ASDALY** | **Change** |
| --- | --- | --- |
| Greenland | 1769(1436.88-2101.49) | -0.19(-0.35--0.01) |
| Monaco | 1483.26(1181.87-1811.68) | 0.37(0.01-0.83) |
| Montenegro | 1343.58(1092.16-1633.36) | 0.11(-0.12-0.4) |
| Hungary | 1220.97(992.75-1496.03) | -0.06(-0.24-0.15) |
| Serbia | 1183.5(919.2-1497.59) | 0.13(-0.14-0.5) |
| Palau | 1071.77(855.72-1337.81) | 0.01(-0.26-0.34) |
| Poland | 1051.75(866.5-1258.55) | -0.14(-0.29-0.03) |
| Northern Mariana Islands | 1021.74(877.24-1161.75) | -0.24(-0.38--0.1) |
| North Macedonia | 1000.4(760.47-1285.24) | 0.26(-0.04-0.63) |
| Bosnia and Herzegovina | 957.92(736.31-1213.42) | -0.03(-0.26-0.24) |
| Nauru | 955.33(605.57-1278.39) | -0.06(-0.3-0.25) |
| Bulgaria | 934.39(730.82-1181.43) | 0.18(-0.08-0.5) |
| Brunei Darussalam | 867.69(768.53-975.37) | -0.12(-0.3-0.11) |
| Solomon Islands | 859.22(460.49-1392.41) | 0.1(-0.22-0.5) |
| Greece | 856.89(806.23-908.45) | -0.11(-0.16--0.05) |
| China | 831.27(699.11-979.99) | 0.09(-0.14-0.37) |
| Georgia | 828.59(684.05-980.38) | -0.03(-0.21-0.19) |
| Romania | 826.92(675.46-1012.38) | 0.1(-0.1-0.35) |
| Netherlands | 825.35(776.81-875.79) | -0.25(-0.3--0.21) |
| Turkey | 814.58(643.77-1017) | -0.19(-0.43-0.13) |
| Uruguay | 813.07(755.22-866.26) | -0.28(-0.33--0.22) |
| Croatia | 811.12(631.78-1038.11) | -0.26(-0.44--0.04) |
| Micronesia | 793.71(469.68-1183.13) | 0.1(-0.27-0.57) |
| Belgium | 793.44(745.75-840.68) | -0.33(-0.37--0.29) |
| Denmark | 786.6(730.36-848.61) | -0.35(-0.4--0.3) |
| Andorra | 783.32(599.49-994.8) | -0.22(-0.47-0.1) |
| France | 780.64(731.05-828.77) | -0.06(-0.12-0) |
| Armenia | 772.08(644.04-919.53) | -0.26(-0.38--0.11) |
| United States of America | 767.35(735.23-791.98) | -0.36(-0.38--0.34) |
| Cuba | 764.93(617.28-936.56) | -0.02(-0.21-0.2) |

## Section 5 The incidence, death, and DALY of liver and TBL cancer in 1990 and 2019 by sex in different agegroups in WHO regions

### *Figure S5-1 African Region*

**A**

**B**

**C**

**D**

**E**

**F**

**G**

**H**

**I**

**J**

**K**

**L**

**Figure S5-1** The incidence, death, and DALY of liver and TBL cancers in 1990 and 2019 by sex in different agegroups in African Region. The incidence (per 100,000 population) of liver cancer in 1990 **(A)** and 2019 **(B)**. The death of (per 100,000 population) liver cancer in 1990 **(C)** and 2019 **(D)**. The DALY of (per 100,000 population) liver cancer in 1990 **(E)** and 2019 **(F)**. The incidence (per 100,000 population) of TBL cancer in 1990 **(G)** and 2019 **(H)**. The death of (per 100,000 population) TBL cancer in 1990 **(I)** and 2019 **(J)**. The DALY of (per 100,000 population) TBL cancer in 1990 **(K)** and 2019 **(L)**.

### *Figure S5-2 Eastern Mediterranean Region*

**A**

**B**

**C**

**D**

**E**

**F**

**G**

**H**

**I**

**J**

**K**

**L**

**Figure S5-2** The incidence, death, and DALY of liver and TBL cancers in 1990 and 2019 by sex in different agegroups in Eastern Mediterranean Region. The incidence (per 100,000 population) of liver cancer in 1990 **(A)** and 2019 **(B)**. The death of (per 100,000 population) liver cancer in 1990 **(C)** and 2019 **(D)**. The DALY of (per 100,000 population) liver cancer in 1990 **(E)** and 2019 **(F)**. The incidence (per 100,000 population) of TBL cancer in 1990 **(G)** and 2019 **(H)**. The death of (per 100,000 population) TBL cancer in 1990 **(I)** and 2019 **(J)**. The DALY of (per 100,000 population) TBL cancer in 1990 **(K)** and 2019 **(L)**.

### *Figure S5-3 European Region*

**A**

**B**

**C**

**D**

**E**

**F**

**G**

**H**

**I**

**J**

**K**

**L**

**Figure S5-3** The incidence, death, and DALY of liver and TBL cancers in 1990 and 2019 by sex in different agegroups in European Region. The incidence (per 100,000 population) of liver cancer in 1990 **(A)** and 2019 **(B)**. The death of (per 100,000 population) liver cancer in 1990 **(C)** and 2019 **(D)**. The DALY of (per 100,000 population) liver cancer in 1990 **(E)** and 2019 **(F)**. The incidence (per 100,000 population) of TBL cancer in 1990 **(G)** and 2019 **(H)**. The death of (per 100,000 population) TBL cancer in 1990 **(I)** and 2019 **(J)**. The DALY of (per 100,000 population) TBL cancer in 1990 **(K)** and 2019 **(L)**.

### *Figure S5-4 Region of the Americas*

**A**

**B**

**C**

**D**

**E**

**F**

**G**

**H**

**I**

**J**

**K**

**L**

**Figure S5-4** The incidence, death, and DALY of liver and TBL cancers in 1990 and 2019 by sex in different agegroups in Region of the Americas. The incidence (per 100,000 population) of liver cancer in 1990 **(A)** and 2019 **(B)**. The death of (per 100,000 population) liver cancer in 1990 **(C)** and 2019 **(D)**. The DALY of (per 100,000 population) liver cancer in 1990 **(E)** and 2019 **(F)**. The incidence (per 100,000 population) of TBL cancer in 1990 **(G)** and 2019 **(H)**. The death of (per 100,000 population) TBL cancer in 1990 **(I)** and 2019 **(J)**. The DALY of (per 100,000 population) TBL cancer in 1990 **(K)** and 2019 **(L)**.

### *Figure S5-5 South-East Asia Region*

**A**

**B**

**C**

**D**

**E**

**F**

**G**

**H**

**I**

**J**

**K**

**L**

**Figure S5-5** The incidence, death, and DALY of liver and TBL cancers in 1990 and 2019 by sex in different agegroups in South-East Asia Region. The incidence (per 100,000 population) of liver cancer in 1990 **(A)** and 2019 **(B)**. The death of (per 100,000 population) liver cancer in 1990 **(C)** and 2019 **(D)**. The DALY of (per 100,000 population) liver cancer in 1990 **(E)** and 2019 **(F)**. The incidence (per 100,000 population) of TBL cancer in 1990 **(G)** and 2019 **(H)**. The death of (per 100,000 population) TBL cancer in 1990 **(I)** and 2019 **(J)**. The DALY of (per 100,000 population) TBL cancer in 1990 **(K)** and 2019 **(L)**.

### *Figure S5-6 Western Pacific Region*

**A**

**B**

**C**

**D**

**E**

**F**

**G**

**H**

**I**

**J**

**K**

**L**

**Figure S5-6** The incidence, death, and DALY of liver and TBL cancers in 1990 and 2019 by sex in different agegroups in Western Pacific Region. The incidence (per 100,000 population) of liver cancer in 1990 **(A)** and 2019 **(B)**. The death of (per 100,000 population) liver cancer in 1990 **(C)** and 2019 **(D)**. The DALY of (per 100,000 population) liver cancer in 1990 **(E)** and 2019 **(F)**. The incidence (per 100,000 population) of TBL cancer in 1990 **(G)** and 2019 **(H)**. The death of (per 100,000 population) TBL cancer in 1990 **(I)** and 2019 **(J)**. The DALY of (per 100,000 population) TBL cancer in 1990 **(K)** and 2019 **(L)**.

## Section 6 The death rate and DALY of liver and TBL cancer in 1990 and 2019 by risk factors in WHO regions

### *Figure S6-1 African Region*

**A**

**B**

**C**

**D**

**E**

**F**

**G**

**H**

**Figure S6-1** The death rate and DALY of liver and TBL cancers in 1990 and 2019 by risk factors in African Region. The death rate (per 100,000 population) of liver cancer in 1990 **(A)** and 2019 **(B)**. The DALY (per 100,000 population) of liver cancer in 1990 **(C)** and 2019 **(D)**. The death rate (per 100,000 population) of TBL cancer in 1990 **(E)** and 2019 **(F)**. The DALY (per 100,000 population) of TBL cancer in 1990 **(G)** and 2019 **(H)**.

### *Figure S6-2 Eastern Mediterranean Region*

**A**

**B**

**C**

**D**

**E**

**F**

**G**

**H**

**Figure S6-2** The death rate and DALY of liver and TBL cancers in 1990 and 2019 by risk factors in Eastern Mediterranean Region. The death rate (per 100,000 population) of liver cancer in 1990 **(A)** and 2019 **(B)**. The DALY (per 100,000 population) of liver cancer in 1990 **(C)** and 2019 **(D)**. The death rate (per 100,000 population) of TBL cancer in 1990 **(E)** and 2019 **(F)**. The DALY (per 100,000 population) of TBL cancer in 1990 **(G)** and 2019 **(H)**.

### *Figure S6-3 European Region*

**A**

**B**

**C**

**D**

**E**

**F**

**G**

**H**

**Figure S6-3** The death rate and DALY of liver and TBL cancers in 1990 and 2019 by risk factors in European Region. The death rate (per 100,000 population) of liver cancer in 1990 **(A)** and 2019 **(B)**. The DALY (per 100,000 population) of liver cancer in 1990 **(C)** and 2019 **(D)**. The death rate (per 100,000 population) of TBL cancer in 1990 **(E)** and 2019 **(F)**. The DALY (per 100,000 population) of TBL cancer in 1990 **(G)** and 2019 **(H)**.

### *Figure S6-4 Region of the Americas*

**A**

**B**

**C**

**D**

**E**

**F**

**G**

**H**

**Figure S6-4** The death rate and DALY of liver and TBL cancers in 1990 and 2019 by risk factors in Region of the Americas. The death rate (per 100,000 population) of liver cancer in 1990 **(A)** and 2019 **(B)**. The DALY (per 100,000 population) of liver cancer in 1990 **(C)** and 2019 **(D)**. The death rate (per 100,000 population) of TBL cancer in 1990 **(E)** and 2019 **(F)**. The DALY (per 100,000 population) of TBL cancer in 1990 **(G)** and 2019 **(H)**.

### *Figure S6-5 South-East Asia Region*

**A**

**B**

**C**

**D**

**E**

**F**

**G**

**H**

**Figure S6-5** The death rate and DALY of liver and TBL cancers in 1990 and 2019 by risk factors in South-East Asia Region. The death rate (per 100,000 population) of liver cancer in 1990 **(A)** and 2019 **(B)**. The DALY (per 100,000 population) of liver cancer in 1990 **(C)** and 2019 **(D)**. The death rate (per 100,000 population) of TBL cancer in 1990 **(E)** and 2019 **(F)**. The DALY (per 100,000 population) of TBL cancer in 1990 **(G)** and 2019 **(H)**.

### *Figure S6-6 Western Pacific Region*

**A**

**B**

**C**

**D**

**E**

**F**

**G**

**H**

**Figure S6-6** The death rate and DALY of liver and TBL cancers in 1990 and 2019 by risk factors in Western Pacific Region. The death rate (per 100,000 population) of liver cancer in 1990 **(A)** and 2019 **(B)**. The DALY (per 100,000 population) of liver cancer in 1990 **(C)** and 2019 **(D)**. The death rate (per 100,000 population) of TBL cancer in 1990 **(E)** and 2019 **(F)**. The DALY (per 100,000 population) of TBL cancer in 1990 **(G)** and 2019 **(H)**.
